# The Dual α-Amidation System in Scorpion Venom Glands

**DOI:** 10.3390/toxins11070425

**Published:** 2019-07-20

**Authors:** Gustavo Delgado-Prudencio, Lourival D. Possani, Baltazar Becerril, Ernesto Ortiz

**Affiliations:** Departamento de Medicina Molecular y Bioprocesos, Instituto de Biotecnología, Universidad Nacional Autónoma de México, Avenida Universidad 2001, Colonia Chamilpa, Cuernavaca, Morelos 62210, Mexico

**Keywords:** amidation, evolution, posttranslational modifications, scorpion, transcriptomics

## Abstract

Many peptides in scorpion venoms are amidated at their C-termini. This post-translational modification is paramount for the correct biological function of ion channel toxins and antimicrobial peptides, among others. The discovery of canonical amidation sequences in transcriptome-derived scorpion proproteins suggests that a conserved enzymatic α-amidation system must be responsible for this modification of scorpion peptides. A transcriptomic approach was employed to identify sequences putatively encoding enzymes of the α-amidation pathway. A dual enzymatic α-amidation system was found, consisting of the membrane-anchored, bifunctional, peptidylglycine α-amidating monooxygenase (PAM) and its paralogs, soluble monofunctional peptidylglycine α-hydroxylating monooxygenase (PHM*m*) and peptidyl-α-hydroxyglycine α-amidating lyase (PAL*m*). Independent genes encode these three enzymes. Amino acid residues responsible for ion coordination and enzymatic activity are conserved in these sequences, suggesting that the enzymes are functional. Potential endoproteolytic recognition sites for proprotein convertases in the PAM sequence indicate that PAM-derived soluble isoforms may also be expressed. Sequences potentially encoding proprotein convertases (PC1 and PC2), carboxypeptidase E (CPE), and other enzymes of the α-amidation pathway, were also found, confirming the presence of this pathway in scorpions.

## 1. Introduction

The order Scorpiones constitutes one of the most ancient lineages within the phylum Arthropoda [1,2]. The key to the ecological success of these arachnids resides in the production of potent venoms used for feeding, defense, and deterring competitors [3,4]. Scorpion venoms are complex mixtures of components, including bioactive peptides with potential therapeutic applications [4], enzymes, metabolites, and most importantly, an arsenal of toxins active on Na^+^, K^+^, Ca^2+^, and Cl^−^ channels [5,6,7,8,9,10]. By altering the normal mechanics of these channels, scorpion toxins unleash systemic havoc in their victims, which can lead to severe envenomation symptoms, including death [11,12]. The venom is produced and secreted by two symmetrical glands located in the last segment of the metasoma, the telson [12]. In these glands, the peptidyl venom components undergo synthesis and maturation, a complex process involving a series of post-translational modifications (PTMs) that result in the biologically active molecules [3]. The most common PTMs found in scorpion venom peptides are the formation of disulfide bridges from pairs of cysteines, proteolytic cleavage, and C-terminal amidation (α-amidation). Amidated toxins and peptides without disulfide bonds (NDBP) are well known in scorpion venoms (Table 1).

Post-translational α-amidation is characteristic of bioactive peptides from many eukaryotic taxa [13]. C-terminal amidation confers on peptides enhanced resilience to degradation by carboxypeptidases, thus increasing their half-lives and decreasing their turnover rates [14]. Moreover, amidation is essential for correct functioning of many mammalian neuropeptides and hormones [15]. Several scorpion toxins have also been shown to require C-terminal amidation for full biological activity, without which, potency is severely reduced [16,17]. C-terminal amidation results in a change with two possible functional implications: the amidated terminal amino acid could be directly involved in molecular recognition events, or the amidation could simply reduce the negative charge of the carboxyl moiety and the peptide as a whole [18].

**Figure 1:**
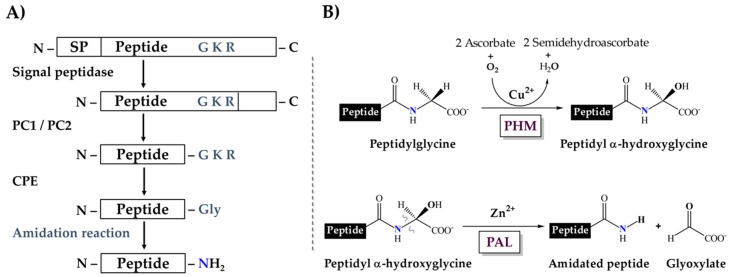
Enzymatic processing of the amidation signal. (**A**) The complete pathway leading to amidation. SP, Signal Peptide; PC1/PC2, proprotein convertases 1/2; CPE, carboxypeptidase E. (**B**) Sequential amidation reaction catalyzed by the PHM and PAL domains.

In general, metazoan amidated peptides are translated as larger polypeptidyl precursors, which contain an amidation signal, a glycine typically followed by one or two basic residues (R-X-Gly-Basic or R-X-Gly-Basic-Basic) and the rest of the propeptide sequence. This signal is first targeted by endoproteolytic proprotein-processing enzymes of the protein convertase family (PCs), resulting in peptides terminated with basic residues, which are substrates for carboxypeptidases that remove those residues from the processing intermediate and expose the C-terminal glycine. This glycine is then further subjected to sequential reactions that amidate the peptide [19,20] (Figure 1A). Two enzymatic activities catalyze these reactions. First, a peptidylglycine α-hydroxylating monooxygenase (PHM, EC 1.14.17.3) catalyzes the hydroxylation of the glycine residue, using ascorbate and molecular oxygen as co-substrates. Then, a peptidyl-α-hydroxyglycine α-amidating lyase (PAL, EC 4.3.2.5) cleaves the hydroxyglycine residue, yielding the amidated product and glyoxylate [21,22] (Figure 1B).

Peptide amidation seems to be common to all metazoans, and PHM and PAL are assumed to have monophyletic origins [23]. However, the way these activities are expressed differs among taxa. For example, in insects *Apis mellifera*, *Drosophila melanogaster* and others, PHM and PAL are encoded by independent genes [24,25,26]. In vertebrates, such as *Bos taurus*, *Rattus norvegicus*, *Xenopus laevis*, *Homo sapiens* and others, a single gene encodes both activities in a bifunctional enzyme comprising a single polypeptide, peptidylglycine α-amidating monooxygenase (PAM) [27,28,29,30]. The same two-domain PAM structure was reported for the gastropod *Aplysia californica* [31]. Curiously, another gastropod, *Lymnaea stagnalis*, produces a zymogen comprising four different PHM domains and a single PAL domain, which is endogenously converted to a mixture of monofunctional isoenzymes [32]. PAM isoforms have been reported in *R. norvegicus*, with up to seven isoforms generated by alternative splicing. These isoforms include configurations with and without internal proteolytic sites, resulting in both independent PHM and PAL, and the bifunctional PAM [27,33]. Among arthropods, independent PHM and PAL, but not the bifunctional PAM, are expressed in insects, as indicated above, whereas both independent and bifunctional enzymes are expressed in crustaceans [26]. No information is available on the α-amidating system of other subphyla, e.g., the chelicerates, and in particular, the arachnids.

Amidated peptides are common in venoms produced by various animals. For example, marine snails of the genus *Conus*, produce a large array of peptidyl toxins (conotoxins), a significant fraction of which are amidated. From the venom ducts of cone snails, cDNAs were cloned that encode bifunctional PAMs. Heterologously expressed PAMs were demonstrated to be active [34]. Although no amidating system has been described in arachnids, the abundance of amidated peptides in their venoms, in particular scorpion venoms, suggests that amidating enzymes are active in their venom glands. The correlation between canonical α-amidation signals in transcripts from different transcriptomic analyses and amidation of the mature encoded peptides, confirmed by biochemical and proteomic analyses [35,36,37,38,39], indicates that the classical PHM plus PAL and/or PAM systems must be present in scorpion venom glands. In this work, the enzymatic amidation system of Old and New World scorpions is assessed by transcriptomic analysis.

## 2. Results

### 2.1. The Dual Enzymatic System for α-Amidation in the Order Scorpiones

**Figure 2:**
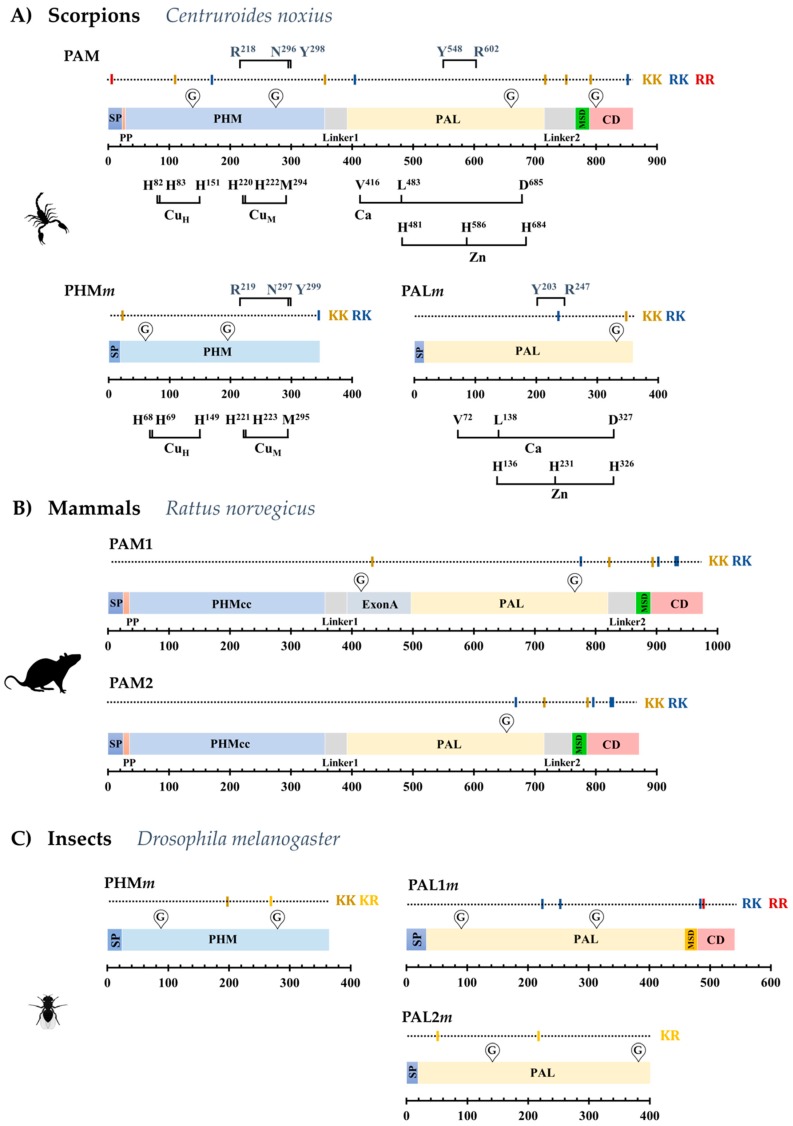
Representative structures of the precursors of (**A**) scorpion (*C. noxius*) bifunctional PAM and mono-functional PHM*m* and PAL*m*; (**B**) mammalian (*R. norvegicus*) PAM-1 and PAM-2 isoforms; (**C**) monofunctional PHM*m*, PAL1*m* and PAL2*m* in *D. melanogaster*. Structural and sequence features are indicated as: SP, Signal peptide; PP, Propeptide; MSD, Membrane Spanning Domain; CD, Cytosolic Domain; KK, RK, KR and RR, putative proprotein convertase cleavage sites at basic dyads; G, predicted glycosylation site; PHMcc, catalytic core of the PHM domain.

We investigated venom gland transcriptomes of 21 scorpion species and the genome of *Centruroides sculpturatus* and identified sequences encoding orthologs of the bifunctional PAM enzyme in 13 of them. Partial sequences for PAM were found in the remaining eight transcriptomes. A 14th complete PAM-coding sequence was recovered by RT-PCR from venom-gland total RNA from the scorpion *Centruroides noxius* (Table 2 and Supplementary Table S2). Complete coding sequences (CDS) from those transcripts translate into proteins of 861–887 amino acids (Supplementary Figure S1). The deduced topology of the scorpion PAM precursor is similar to that of the PAM-2 isoform described for *Rattus norvegicus* (Figure 2A,B). A signal peptide sequence (SP) for secretion is followed by a short propeptide (PP) region, a PHM domain, a linker sequence (Linker 1), a PAL domain, a second linker sequence (Linker 2), a membrane spanning domain (MSD), and a cytosolic domain (CD) (Figure 2A). The rat PAM-2 isoform lacks the Exon A-encoded linker region with respect to the rat PAM-1 isoform. This extra region contains an endoproteolytic site which, after processing, cleaves the PHM and PAL monofunctional enzymes into separate polypeptides. This Exon-A-encoded region has been described only for vertebrates [23], and has no equivalent sequence in the scorpion PAM (Supplementary Figure S2). It is notable that although the scorpion PAM lacks this region, two putative endoproteolytic sites are still present in the scorpion PAM sequence (Figure 2A). The first site, defined by a lysine dyad (KK), is located between the PHM and PAL domains, and is proposed to delimit the PHM domain. The second site, located between the PAL sequence and the MSD, is also defined by a KK dyad, and if subjected to post-translational processing, would liberate a soluble PAL enzyme from the MSD and CD domains. Thus, the scorpion bifunctional PAM enzyme could be post-translationally processed to generate independent, soluble PHM and PAL enzymes.

Shorter transcripts encoding the monofunctional PHM and PAL enzymes (PHM*m* and PAL*m*) were also identified in most of the analyzed scorpion transcriptomes/genome (Table 2 and Supplementary Table S2). The encoded proteins are 345–350 amino acids long (PHM*m*) and 356–366 (PAL*m*) (Supplementary Figures S3 and S4). Topologies of the monofunctional enzymes are similar to those of the PHM and PAL-2 isoforms from *D. melanogaster* (Figure 2C). The proproteins include a SP and the catalytic domain. No MSD and CD domains are detected; therefore, the monofunctional enzymes are predicted to be soluble.

Key residues involved in catalysis and metal coordination are conserved in both scorpion amidation systems (Figure 2A and Supplementary Figures S5 and S6), suggesting that those enzymes are probably functional. The percentage of sequence identity between homologous domains of the bifunctional and independent enzymes for each species are indicated in Supplementary Table S3. As an example, for *C. noxius*, the percentage of identity between the PAM subdomains and the PHM*m* and PAL*m* are 29.8% and 32.5%, respectively.

Sequences encoding other components of the α-amidation pathway were also sought among available scorpion transcriptomic/genomic sequences. Transcripts encoding orthologs of proprotein convertases 1 and 2 (PC1 and PC2) and carboxypeptidase E (CPE), enzymes that operate upstream in the α-amidation pathway (Figure 1A), were also found, as well as their genes in the *Centruroides sculpturatus* genome (Table 2, Supplementary Table S2), reinforcing the notion of a conserved α-amidation pathway in scorpions.

These results indicate that in scorpions, a dual enzymatic system for α-amidation is responsible for the amidation of venom peptides. Transcripts for both the bifunctional PAM and the monofunctional PHMm and PALm are present in scorpion venom glands. Among arthropods, a similar dual system is present in crustaceans, but not in insects [26].

### 2.2. The PAM-, PHMm- and PALm-Coding Genes

**Figure 3:**
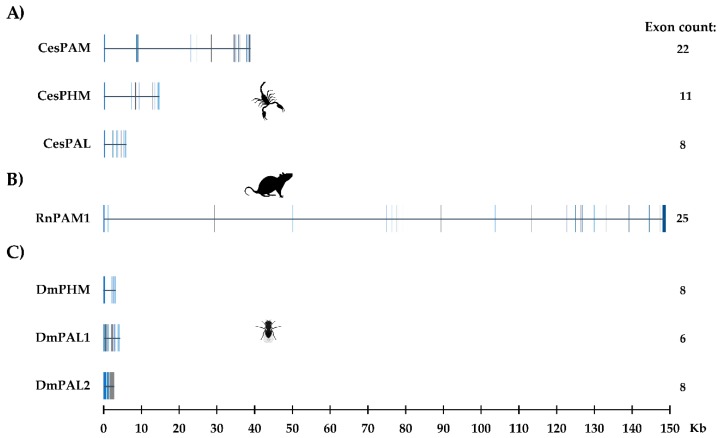
The structure of genes encoding the amidating enzymes of (**A**) *C. sculpturatus* (CesPAM, CesPHM and CesPAL); (**B**) *R. norvegicus* (PAM1); (**C**) *D. melanogaster* (DmPHM, DmPAL1 and DmPAL2). Exons are indicated as vertical blocks.

The search for genomic sequences in *C. sculpturatus* using blastn showed that separate genes encode the bifunctional and monofunctional enzymes, demonstrating that they are encoded by paralogous genes and are not the result of alternative splicing, a phenomenon reported in the generation of isoforms in *R. norvegicus* [27,33]. Figure 3A shows the structure of the genes for the PAM, PHM*m* and PAL*m* enzymes in *C. sculpturatus*, including their sizes, exon numbers and distributions. The structures of the rat PAM and fruit fly PHM and PAL genes are also shown for comparison (Figure 3B,C).

### 2.3. Phylogenetic Reconstruction of Amidating Enzymes of Arachnids

**Figure 4:**
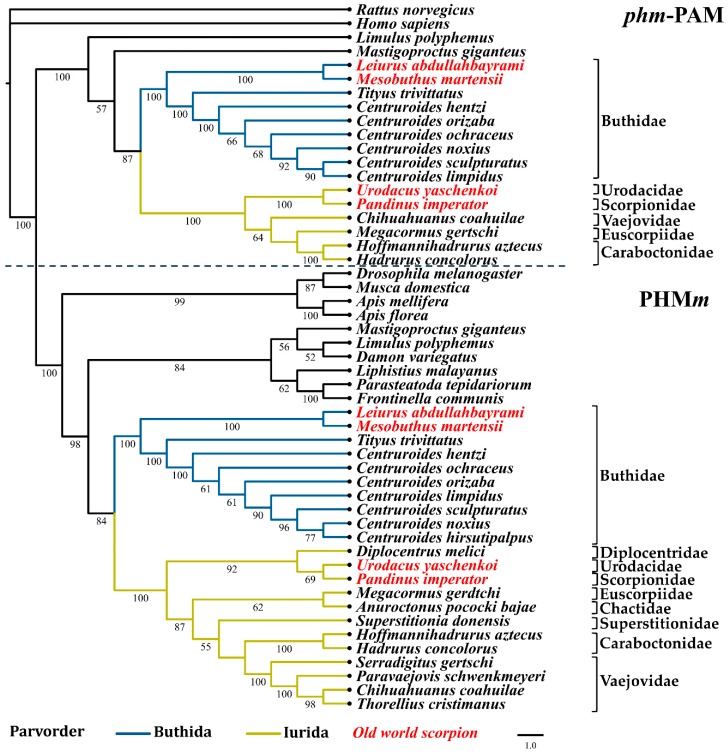
Reconstructed evolutionary history of the phm-PAM and PHM*m* domains. Maximum likelihood analyses were performed with nucleotide sequences corresponding to the respective domains. Numbers under the nodes indicate the values of ultrafast bootstrap (UFBoot) (only branches with values higher that 50 are shown).

**Figure 5:**
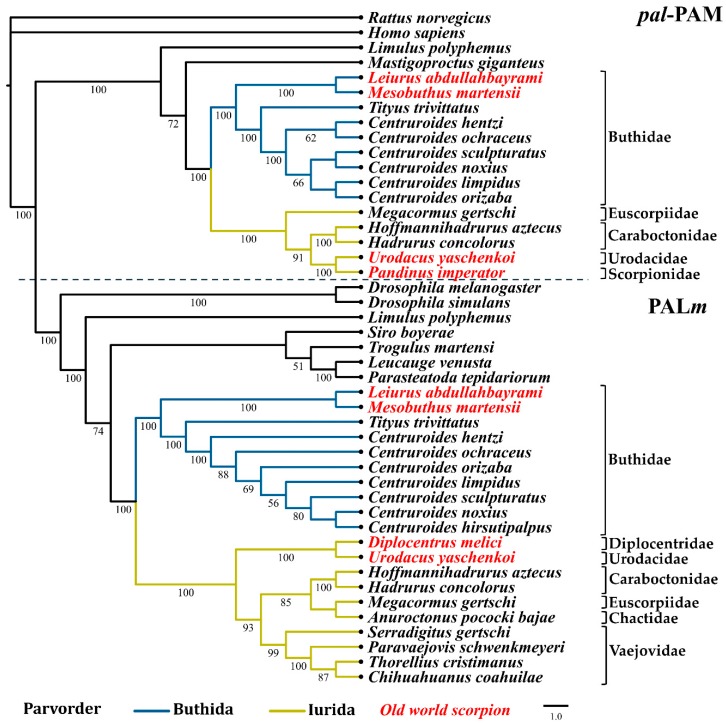
Reconstructed evolutionary history of the pal-PAM and PAL*m* domains. Maximum likelihood analyses were performed with the nucleotide sequences corresponding to the respective domains. Number under the nodes indicate the values of ultrafast bootstrap (UFBoot) (only branches with values higher that 50 are shown).

Phylogenomic analyses have proposed two basal branches from which all scorpions have descended (parvorders Buthida and Iurida) [1,40,41]. Maximum likelihood analyses with the nucleotide sequences of the PHM and PAL domains from the PAM (designated as *phm*-PAM and *pal*-PAM in these analyses, respectively) and the PHM*m* and PAL*m* enzymes, show a correlation between the phylogeny of these enzymes and the phylogeny of the scorpion families from which they originate [40]. Figure 4; Figure 5 show the two main clades in which the sequences of the *phm*-PAM and *pal*-PAM are separated from the sequences PHM*m* and PAL*m*, respectively. Within those clades, there is a clear divergence between sequences from species belonging to the family Buthidae (parvorder Buthida) and sequences from species belonging to families of the parvorder Iurida (Vaejovidae, Caraboctonidae, Euscorpiidae, Chactidae, Superstitionidae, Diplocentridae, Urodacidae, Scorpionidae). Within the family Buthidae, sequences from Old World scorpions *Leiurus abdullahbayrami* (Turkey) and *Mesobuthus martensii* (Eastern Asian countries) are placed in an independent, supported clade that precedes the clade of New World species. The sequences from *Tityus trivittatus* (from the south-central part of South America, Argentina, and Brazil) are in independent supported clades with respect to those of the genus *Centruroides* (distributed in Central America, the Caribbean, and North America). The variable numbers of sequences recovered from different transcriptomes, limits comparative analyses of the catalytic domains, though a consistent topology for the phylogenetic trees is observed. Sequences putatively encoding a dual amidation system, as in scorpions, were also found in other arachnids, including members of the orders Araneae (*Liphistius malayanus*, *Frontinella communis*, *Parasteatoda tepidariorum*, *Leucauge venusta*), Opiliones (*Siro boyerae, Trogulus martensi*) Ricinulei (*Ricinoides atewa*), and the xiphosuran, *Limulus polyphemus* (recently placed within the class Arachnida [42]), among others (Supplementary Table S1). This indicates that the same dual α-amidation system is also employed by other arachnids.

## 3. Discussion

Venom gland transcriptomic analyses performed with representative scorpion families from both the Old and New Worlds have shown the enormous diversity of compounds that comprise these important biofluids [4]. Together with available biochemical information on scorpion venom components, sequences of many transcripts indicate that amidation is one of the most common PTMs of scorpion venom peptides. The discovery of canonical amidation signals in the translated sequences suggested that a conserved α-amidation system might be present in scorpion venom glands to convert propeptides into shorter, amidated, mature peptides. In this work, transcripts encoding the relevant components of this pathway are described, confirming that a dual amidation system, including a bifunctional PAM enzyme and individual non-membrane bound PHM*m* and PAL*m* is employed. Genes for this dual system were found in the genome of *C. sculpturatus*, demonstrating than the bifunctional and the monofunctional enzymes are encoded by independent genes and are not the result of alternative splicing. Paralogs involved in various developmental processes and cellular functions within the orders Scorpiones and Araneae arose as a consequence of a genome duplication in the common ancestor of scorpions and spiders [43,44]. Given the importance of amidation in peptide signaling and the functionality of toxins and other amidated venom peptides, it is not surprising that both amidation enzyme systems were retained in this lineage of venomous arachnids, where they evolved to target specific substrates, or to be expressed in particular cell types or physiological conditions.

Together with conserved functional residues for cation coordination and enzymatic activity, the scorpion PAM sequence contains all the structural elements for generation of a membrane-anchored protein. However, the sequence of the bifunctional PAM contains putative endoprotease cleavage sites (dyads of basic amino acids), which are normally targeted by proprotein convertases, flanking the catalytic domains. This means that the PAM proprotein could in principle be processed to the complete membrane-bound two-domain enzyme or it could be post-translationally cleaved by convertases to render soluble monofunctional domains. The presence of transcripts encoding convertases in the scorpion venom glands, also described in this work, reinforces this possibility. Whether both the two-domain PAM and the PAM-derived monofunctional enzymes coexist in the venom gland remains to be established. We expect that the soluble PHM*m* and PAL*m*, as well as the putative PAM-derived soluble isoforms, are secreted by the venom glands into the venom. This has been confirmed, at least for PHM*m* with liquid chromatography-mass spectrometry (LC-MS/MS) in scorpion venom proteomic analyses. Although it is not clear what additional functions they might have in scorpion venom, it is known that the bovine PAM enzyme is capable of catalyzing three alternative reactions: sulfoxidation, N-dealkylation of amines and O-dealkylation [45]. This raises the possibility of finding new natural substrates for this set of enzymes and taking advantage of their catalytic capacities for synthesis or chemical modification of molecules of biotechnological interest.

Other proteomic analyses have confirmed the presence of putative amidating enzymes in arachnid venoms. One of these sequences was reported as a PAM from the spider *Cupiennius salei* (annotated as PAM_CUPSA [MH766628]) [46]. However, a rigorous sequence analysis demonstrates that this sequence is not from a PAM ortholog, but a monofunctional PHM*m*. Similarly, for the scorpion *Tityus obscurus*, a sequence reported as a PAM (GenBank: JAT91064) [38], shares 87% sequence identity with the PHM*m* from *T. trivittatus*, as reported here, and is therefore also a PHM*m*. A third report found a PHM*m* sequence in transcriptomic and proteomic analyses of the scorpion *C. hentzi* (annotated as GFWZ01000197.1 TSA: *Centruroides hentzi* Chent_MonoO transcribed RNA sequence) [47]. Sequences encoding orthologs of PHM*m* were also identified in venoms of *Centruroides limpidus*, *Centruroides hirsutipalpus* and *Superstitionia donensis* (data not shown). Therefore, this constitutes the first report of the monofunctional PAL*m* and the bifunctional PAM enzymes from any arachnid, and demonstrates that a conserved, functional dual α-amidation system is present in scorpion venom glands, as well as in other arachnids.

## 4. Materials and Methods

### 4.1. Sequence Data and Transcriptome Assembly

Previously reported transcriptomic analyses from venom glands of the scorpion species C. limpidus, Paravaejovis schwenkmeyeri, Urodacus yaschenkoi, Thorellius cristimanus (reported as T. atrox), Serradigitus gertschi, S. donensis, and Megacormus gertschi [37,48,49,50,51,52,53] were used to obtain relevant sequence information. Complementary sequence information was obtained from other unpublished transcriptomes for the species Centruroides noxius, C. orizaba, C. ochraceus, C. hirsutipalpus, T. trivittatus, L. abdullahbayrami, Hoffmannihadrurus aztecus, Hadrurus concolorus, Anuroctonus pococki bajae, Chihuahuanus coahuilae and Diplocentrus melici. Publicly available reads from massive transcriptome analyses of other species were assembled de novo and also used (M. martensii SRR3061379, Pandinus imperator SRR1721600, C. hentzi SRR6041834/SRR6041835; external groups are shown in Supplementary Table S1). Assembly was performed using Trinity 2.0.3 [54] with previously reported parameters [37]. Genomic sequences from C. sculpturatus (BioProject: PRJNA168116) were obtained from NCBI. Sequence information from 22 different scorpion species, belonging to nine of the 20 recognized scorpion families [55] was used in this work.

### 4.2. Identification and Annotation of Amidating Enzymes in Scorpions and Related Organisms

Sequences putatively encoding PAM, PHM and PAL homologs were identified in transcriptomes using tBLASTn, with the sequence of the *R. norvegicus* PAM (Uniprot, P14925) as query. Recovered nucleotide sequences were translated with the ExPASy server [56]. The presence and organization of characteristic domains was evaluated with NCBI-CDART [57] in accordance with [23]. Other sequence hallmarks were identified: the signal peptide (SP) with SignalP 4.1 and Phobius [58,59], the propeptide region (Pp) with ArachnoServer v. 3.0 [60] and the transmembrane domain with the TMHMM server v. 2.0 [61]. Identification and delimitation of the catalytic domains and the residues involved in metal coordination and disulfide formation was manually performed by sequence alignment with the reference *R. norvegicus* PAM (Uniport P14925). Potential glycosylation sites were predicted with the NetNGlyc 1.0 Server (http://www.cbs.dtu.dk/services/NetNGlyc/). The annotation of each determined sequence can be found in Supplementary Table S1. The sequences were submitted to the European Nucleotide Archive (ENA) under project PRJEB32831.

### 4.3. Amplification and Cloning of the PAM Sequence from Centruroides noxius

Total RNA was extracted from the telson of a single female *C. noxius* using an SV Total RNA Isolation System kit (Promega Corporation, Madison, WI, USA). cDNA was amplified with a First Strand cDNA Synthesis Kit for RT-PCR (AMV) (Roche, Basel, Switzerland). Primers, Cen-Fw3 (5′-GAT CTT GTA AAC GGC GTA TTT CCC TT-3′) and Cen-Rv4 (5′-CCG ATA TCC TCC CAA CCA TCC TTT C-3′), were designed from the consensus of the PAM sequences from two scorpions of the genus *Centruroides* (*C. limpidus* and *C. orizaba*). Amplification conditions were 3 min at 96 °C, followed by 30 cycles of 3 sec at 96 °C, 1 min at 56 °C and 2 min at 68 °C, plus a final step of 5 min at 68 °C. A recombinant Pfu polymerase produced in-house was used. The PCR product was purified with the QIAQuick Gel extraction Kit (QIAGEN GmbH, Hilden, Germany), ligated into an EcoRV-digested pBluescript II KS(+) vector, and electroporated to electrocompetent DH5α *Escherichia coli* cells. Positive clones were selected with the blue/white system by growing the cells in X-Gal/IPTG-complemented LB/ampicillin medium. Plasmids were prepared by alkaline lysis and submitted to sequencing with the primers T7-Like (5′-GCG TAA TAC GAC TCA CTA TA-3′), T3-Like (5′-CTC ACT AAA GGG AAC AAA AGC-3′), Cen-In1 (5′-CTC GTT GCT TAG ATA TAG AGA-3′), Cen-In2 (5′-ACA TCA GTC AAC CAA ACA-3′) and Cnox-In3 (5′-ATT GAT GCT GAT GAT GCC TA-3′).

### 4.4. Multiple Alignments and Phylogeny Reconstruction of PAM, PHM, and PAL

Phylogenetic reconstruction of the PAM enzyme and its two catalytic domains *phm*-PAM and *pal*-PAM (with the suffix ‘-PAM’ used to differentiate them from those of the monofunctional enzymes), and of the independent enzymes PHM*m* and PAL*m* (with the suffix ‘*m*’, for ‘monofunctional’) was performed using the maximum likelihood (ML) method with nucleotide sequences. Additional sequences from phylogenetically related organisms (external groups) were obtained from NCBI or assembled from transcriptome raw reads deposited at SRA-NCBI. All sequences were aligned with MAFFT v7.407 [62]. The best substitution model (GTR+F+I+G4) and the ML analysis were evaluated with IQ-TREE v1.6.9 [63,64], using the ultrafast bootstrap method (UFBoot2) [65] with 10,000 replicates.

### 4.5. Genomic Organization of Scorpion PAM, PHM, and PAL

Genome sequences of *C. sculpturatus* (NCBI:txid218467) corresponding to the amidating enzymes were recovered from NCBI using BLASTn, with the nucleotide sequences for PAM, PHM, and PAL from *C. limpidus* as queries. Identification of introns and exons was performed with the Splign utility [66].

## Figures and Tables

**Table 1:** Diversity of amidated peptides from scorpion venoms. Signal peptides are underlined. Mature peptides are in bold upper-case letters. Propeptides are italicized and amidation signals are shown in red.

Type	Peptide	Uniprot	Precursor Sequence	C-terminus
Sodium toxins	AaH2	P01484	MNYLVMISLALLFVTGVES **VKDGYIVDDVNCTYFCGRNAYCNEECTKLKGESGYCQWASPYGNACYCYKLPDHVRTKGPGRCH** * **GR** *	**H-NH_2_**
	LqhIT2	Q26292	MKLLLLLIVSASMLIESLVNA **DGYIKRRDGCKVACLIGNEGCDKECKAYGGSYGYCWTWGLACWCEGLPDDKTWKSETNTCG** * **GKK** *	**G-NH_2_**
	BmKITa	Q9XY87	MKLFLLLLISASMLIDGLVNA **DGYIRGSNGCKVSCLWGNEGCNKECRAYGASYGYCWTWGLACWCQGLPDDKTWKSESNTCG** * **GKK** *	**G-NH_2_**
	Cn2	P01495	LLIITACLALIGTVWA **KEGYLVDKNTGCKYECLKLGDNDYCLRECKQQYGKGAGGYCYAFACWCTHLYEQAIVWPLPNKRCS** * **GK** *	**S-NH_2_**
	Css4	P60266	MNSLLMITACLALVGTVWA **KEGYLVNSYTGCKFECFKLGDNDYCLRECRQQYGKGSGGYCYAFGCWCTHLYEQAVVWPLPNKTCN** * **GK** *	**N-NH_2_**
	CsEI	P01491	MNSLLMITACLVLIGTVWA **KDGYLVEKTGCKKTCYKLGENDFCNRECKWKHIGGSYGYCYGFGCYCEGLPDSTQTWPLPNKTC** * **GKK** *	**C-NH_2_**
	CsEv3	P01494	MNSLLMITACLFLIGTVWA **KEGYLVNKSTGCKYGCLKLGENEGCDKECKAKNQGGSYGYCYAFACWCEGLPESTPTYPLPNKSC** * **GKK** *	**C-NH_2_**
	Ts1	P15226	MKGMILFISCLLLIGIVVEC **KEGYLMDHEGCKLSCFIRPSGYCGRECGIKKGSSGYCAWPACYCYGLPNWVKVWDRATNKC** * **GKK** *	**C-NH_2_**
	Ts3	P01496	LVVVCLLTAGTEG **KKDGYPVEYDNCAYICWNYDNAYCDKLCKDKKADSGYCYWVHILCYCYGLPDSEPTKTNGKCKS** * **GKK** *	**S-NH_2_**
Potassium toxins	NTx	P08815	MKAFYGILIILLFCSMFNLNES **TIINVKCTSPKQCSKPCKELYGSSAGAKCMNGKCKCYNN** * **G** *	**N-NH_2_**
	BmKTX	Q9NII7	MKVFFAVLITLFICSMIIGIHG **VGINVKCKHSGQCLKPCKDAGMRFGKCINGKCDCTPK** * **G** *	**K-NH_2_**
	CoTx1	O46028	MEGIAKITLILLFLFVTMHTFANWNTEA **AVCVYRTCDKDCKRRGYRSGKCINNACKCYPY** * **GK** *	**Y-NH_2_**
	OcKTx5	Q6XLL5	MNAKFILLLVLTTMMLLPDTKG **AEVIRCSGSKQCYGPCKQQTGCTNSKCMNKVCKCYGC** * **G** *	**C-NH_2_**
	OcKTx1	Q6XLL9	MNAKFILLLLVVATTMLLPDTQG **AEVIKCRTPKDCAGPCRKQTGCPHGKCMNRTCRCNRC** * **G** *	**C-NH_2_**
Non disulfide bridged peptides	IsCT	Q8MMJ7	MKTQFAILLVALVLFQMFAQSDA **ILGKIWEGIKSLF** *GKRGLSDLDGLDELFDGEISKADRDFLRELMR*	**F-NH_2_**
	BmKb1	Q718F4	MEIKYLLTVFLVLLIVSDHCQA **FLFSLIPSAISGLISAFK** *GRRKRDLNGYIDHFKNFRKRDAELEELLSKLPIY*	**K-NH_2_**
	Hp1090	P0DJ02	MKTQFAIFLITLVLFQMFSQSDA **IFKAIWSGIKSLF** *GKRGLSDLDDLDESFDGEVSQADIDFLKELMQ*	**F-NH_2_**
	IsCT2	Q8MTX2	MKTQFAILLVALVLFQMFAQSEA **IFGAIWNGIKSLF** *GRRALNNDLDLDGLDELFDGEISQADVDFLKELMR*	**F-NH_2_**
	VAMP-2	E4VP07	MKSQTFFLLFLVVFLLAITQSEA **IFGAIAGLLKNIF** *GKRSLRDMDTMKYLYDPSLSAADLKTLQKLMENY*	**F-NH_2_**

**Table 2:** Enzymes of the α-amidation pathway detected in scorpions.

Family	Species	PAM	PHM	PAL	PC1	PC2	CPE
Buthidae	*Centruroides sculpturatus*	◼-◼	◼	◼	⬤	⬤	⬤
	*Centruroides hentzi*	◼-◼	◼	◼	⬤		⬤
	*Centruroides noxius ^a^*	◼-◼	◼	◼	⬤		⬤
	*Centruroides limpidus ^b^*	◼-◼	◼	◼	⬤	⬤	⬤
	*Centruroides orizaba*	◼-◼	◼	◼	⬤	⬤	⬤
	*Centruroides ochraceus*	◼-◼	◼	◼	⬤	⬤	⬤
	*Centruroides hirsutipalpus*	✓	◼	◼	⬤		⬤
	*Tityus trivittatus*	◼-◼	◼	◼	⬤	⬤	⬤
	Leiurus abdullahbayrami *	◼-◼	◼	◼	⬤	⬤	⬤
	Mesobuthus martensii *	◼-◼	◼	◼			
Vaejovidae	*Thorellius cristimanus*	◼-◼	◼	◼	⬤	⬤	⬤
	*Paravaejovis schwenkmeyeri*	✓	◼	◼		⬤	⬤
	*Chihuahuanus coahuilae*	◼-◼	◼	◼	⬤	⬤	⬤
	*Serradigitus gertschi*	✓	◼	◼			
Caraboctonidae	*Hoffmannihadrurus aztecus*	◼-◼	◼	◼			
	*Hadrurus concolorus*	◼-◼	◼	◼		✓	⬤
Euscorpiidae	*Megacormus gertschi*	◼-◼	◼	◼			
Chactidae	*Anuroctonus pococki bajae*	✓	◼	◼			
Superstitionidae	*Superstitionia donensis*	✓	◼	✓		✓	
Diplocentridae	*Diplocentrus melici*	◼-◼	◼	◼		⬤	
Urodacidae	Urodacus yaschenkoi *	◼-◼	◼	◼	⬤	✓	⬤
Scorpionidae	*Pandinus imperator* *	◼-◼	◼			⬤	

(◼**-**◼, ◼ and ◼): Complete PAM, PHM*m* and PAL*m* sequences; (◼**-**◼): Partial PAM sequences with 93% or more of the sequence determined; (⬤, ⬤, ⬤): Complete PC1, PC2 and CPE sequences; (⬤, ⬤, ⬤): PC1, PC2 and CPE sequences with more than 50% of the sequence determined; (✓): Partial sequences with less than 50% of the estimated total sequence determined; ^a^ PAM sequence amplified by PCR; ^b^ PAM sequence verified by DNA sequencing; * Old World scorpion. The tblast and blastn algorithms were used to identify sequences in the local scorpion transcriptomic databases, with an e-value of 1 × 10^−6^. Empty spaces indicate that no sequences were identified in those transcriptomes.

## Supplementary Materials

The following are available online at https://www.mdpi.com/2072-6651/11/7/425/s1: Figure S1: Schematic alignment of PAM sequences with >90% of the estimated sequence determined, Figure S2: Schematic alignment of PAM1 and PAM2 isoforms from *R. norvegicus* and the completely sequenced scorpion PAM, Figure S3: Schematic alignment of the PHM*m* sequences found in 22 analyzed scorpion transcriptomes, Figure S4: Schematic alignment of the 20 PAL*m* sequences found in 22 analyzed scorpion transcriptomes, Figure S5: Sequence alignment of PHM domains, Figure S6: Sequence alignment of PAL domains, Table S1: Nomenclature of transcripts in various scorpion species, Table S2: Sequence conservation between catalytic domains of the bifunctional and monofunctional enzymes by species (% of identity), Table S3: External groups used for phylogenetic reconstruction of the evolutionary history of the functional domains.
