# Supplementary material for: The Dual α-Amidation System in Scorpion Venom Glands
Source: Toxins (Basel). 2019 Jul 20;11(7):425. doi: 10.3390/toxins11070425 (PMC6669573; doi:10.3390/toxins11070425)
Supplement: Supplementary file 1 [file toxins-11-00425-s001.zip › Delgado-Prudencio Suplementary_Table_S1_R2 v1.docx]

**Supplementary Table S1.** Nomenclature of transcripts in various scorpion species. Only transcripts with completely assembled sequences were annotated and reported.

| **Family** | **Species** | **PAM** | **PHM** | **PAL** | **PC1** | **PC2** | **CPE** |
| --- | --- | --- | --- | --- | --- | --- | --- |
| Buthidae | *Centruroides sculpturatus* | CesEnzPAM01 | CscEnzPHM01 | CscEnzPAL01 | CscEnzPC101 | CscEnzPC201 | CscEnzCPE01 |
|  | *Centruroides hentzi* | CheEnzPAM01 | CheEnzPHM01 | CheEnzPAL01 | CheEnzPC101 |  | CheEnzCPE01 |
|  | *Centruroides noxius* | CnoEnzPAM01 | CnoEnzPHM01 | CnoEnzPAL01 |  |  |  |
|  | *Centruroides limpidus* | CliEnzPAM01 | CliEnzPHM01 | CliEnzPAL01 | CliEnzPC101 | CliEnzPC201 | CliEnzCPE01 |
|  | *Centruroides orizaba* | CorEnzPAM01 | CorEnzPHM01 | CorEnzPAL01 | CorEnzPC101 | CorEnzPC201 | CorEnzCPE01 |
|  | *Centruroides ochraceus* | CocEnzPAM01 | CocEnzPHM01 | CocEnzPAL01 |  |  | CocEnzCPE01 |
|  | *Centruroides hirsutipalpus* |  | ChiEnzPHM01 | ChiEnzPAL01 |  |  | ChiEnzCPE01 |
|  | *Tityus trivittatus* | TtrEnzPAM01 | TtrEnzPHM01 | TtrEnzPAL01 | TtrEnzPC101 | TtrEnzPC201 | TtrEnzCPE01 |
|  | *Leiurus abdullahbayrami* | LabEnzPAM01 | LabEnzPHM01 | LabEnzPAL01 | LabEnzPC101 | LabEnzPC201 | LabEnzCPE01 |
|  | *Mesobuthus martensii* | MmaEnzPAM01 | MmaEnzPHM01 | MmaEnzPAL01 |  |  |  |
| Vaejovidae | *Thorellius cristimanus* |  | TcrEnzPHM01 | TcrEnzPAL01 | TcrEnzPC101 | TcrEnzPC201 | TcrEnzCPE01 |
|  | *Paravaejovis schwenkmeyeri* |  | PscEnzPHM01 | PscEnzPAL01 |  | PscEnzPC201 | PscEnzCPE01 |
|  | *Chihuahuanus coahuilae* |  | CcoEnzPHM01 | CcoEnzPAL01 | CcoEnzPC101 |  | CcoEnzCPE01 |
|  | *Serradigitus gertschi* |  | SgeEnzPHM01 | SgeEnzPAL01 |  |  |  |
| Caraboctonidae | *Hoffmannihadrurus aztecus* | HazEnzPAM01 | HazEnzPHM01 | HazEnzPAL01 |  |  |  |
|  | *Hadrurus concolorus* | HcoEnzPAM01 | HcoEnzPHM01 | HcoEnzPAL01 |  |  | HcoEnzCPE01 |
| Euscorpiidae | *Megacormus gertschi* | MgeEnzPAM01 | MgeEnzPHM01 | MgeEnzPAL01 |  |  |  |
| Chactidae | *Anuroctonus pococki bajae* |  | ApoEnzPHM01 | ApoEnzPAL01 |  |  |  |
| Superstitionidae | *Superstitionia donensis* |  | SdoEnzPHM01 |  |  |  |  |
| Diplocentridae | *Diplocentrus melici* |  | DmeEnzPHM01 | DmeEnzPAL01 |  | DmeEnzPC201 |  |
| Urodacidae | *Urodacus yaschenkoi* | UyaEnzPAM01 | UyaEnzPHM01 | UyaEnzPAL01 | UyaEnzPC101 |  | UyaEnzCPE01 |
| Scorpionidae | *Pandinus imperator* | PimEnzPAM01 | PimEnzPHM01 |  |  | PimEnzPC201 |  |
