# Supplementary material for: The Dual α-Amidation System in Scorpion Venom Glands
Source: Toxins (Basel). 2019 Jul 20;11(7):425. doi: 10.3390/toxins11070425 (PMC6669573; doi:10.3390/toxins11070425)
Supplement: Supplementary file 1 [file toxins-11-00425-s001.zip › Delgado-Prudencio Supplementary_Figure_S5_R2 v4.docx]

**10 20 30 40 50 60 70 80 90 100**

**....|....|....|....|....|....|....|....|....|....|....|....|....|....|....|....|....|....|....|....|**

**R.norvegicus** -FKET--TRSFSNECLGTIGPVTPLDASDFALDIRMPGVTPKESDTYFCMSMRLPVDEEAFVIDFKPRASMDTVHHMLLFGCNMPSSTG----SY-WFC-

*phm*-PAM

PHM*m*

C.sculpturatus --------------------AEP.MQKDH..IN.N..N.K.TKH.S.I.TTK.IDA.-D.YI.Q.E.S.DALRA...I.....SLY.PNYIYPEH-.N.-

C.hentzi --------------------AEP.MQKDH..IN.Y..N.K.TKH.S.I.TTK.IDA.-D.YI.Q.E.S.DALRA...I.....SLY.PNYIYPEH-.N.-

C.noxius --------------------AEP.MQKDH..IN.N..N.K.NKH.S.I.TTK.IDA.-D.YI.Q.E.S.DALRA...I.....SLY.PNYIYPEH-.N.-

C.limpidus --------------------AEP.MQKDH..IN.N..N.K.TKH.S.I.TTK.IDA.-D.YI.Q.E.S.DALRA...I.....SLY.PNYIYPEH-.N.-

C.orizaba --------------------AEPSMQKDH..IN.N..N.K.TKH.S.I.TTK.IDA.-D.YI.Q.E.S.DALRA...I.....SLY.PNYIYPEH-.N.-

C.ochraceus --------------------AEP.MQKDH..IN.N..N.K.TKH.S.I.TTK.IDA.-D.YI.Q.E.S.DALRA...I.....SLY.PNYIYPEH-.N.-

T.trivittatus --------------------AEP.VQKDH..VN.N..N.K.T.H.S.I.TTK.IDI.-D.YI.N.E.S.DALRS.........SLYTPSYIYPE.-.N.-

L.abdullahbayrami --------------------AEPVTHNDH.VMN.S..N.R.TNH.S.I.TTK..DP.-D.YI.Q.E.S.DALKA........SSLYTASYMYPE.-.K.-

M.martensii --------------------AEPVIHNDH.VMN.S..N.R.INH.S.I.TTK..DP.-D.YI.Q.E.S.DALKA........SSLYTASYMYPE.-.K.-

H.aztecus --------------------AEP.VYTET.TMN.S..N.K.LTD.S.I.TAKHIGSG-S.YITR.E.Y.DVHRA........EELDNLDHLYPH.-.N.-

H.concolorus --------------------AEP.VYTEN.TMN.S..N.K.LTD.S.I.TAK.IGSG-S.YITR.E.Y.DVHRA........EELDNLDHLYPH.-.N.-

M.gertschi --------------------AEP.VYTQN.IMN.S..D.K.SIA.S.I.TAK..SSG-NS.ITG.E.Y.DVQRA...V....EELDNHDHLYPHH-.N.-

U.yaschenkoi --------------------AEP.VYTEN.IMN.S..N.R.LTD.S.I.TARH.GSG-N.YITH.E.H.DVHR.........SALDNHEHLYPNH-.N.-

P.imperator --------------------AER.VYTEN..IN.S..H.R.STP.S.I.TAKHISSG-N.YITR.E.H.DVHR.........SDLDNHERLYPYH-.N.-

T.atrox* --------------------AEP.VYTEN.IMN.S..D.K.STP.S.I.TAKH.GSG-N.YITR.E.Y.DVHRA........EELNNHDHLYPNH-.N.-

C.coahuilae* --------------------AEP.VYTEN.IMN.S..D.K.STP.S.I.TAKH.GSG-N.YITR.E.Y.DVHRA........EELNNRDHLYPNH-.N.-

D.melici* --------------------AEQ.VYTQN.VMN.S..N.R.LTA.S.I.TAKH.GSG-N.YITQ.E.H.DAHR....V.....ELYTNEHIYPHH-.N.-

**D.melanogaster** LV..GDYQN.LYQQN.----ESNSATGATASFPFL..N.S.QTP.L.L.TPIKVDPTTTYYIVG.N.N.T.N.A.....Y..GE.G-----TSKTT.N.G

C.sculpturatus --------------------------FNYKK.PLL..D.Q..QKE..L.VAYKMNKN.HENIVK.E.N.T.HVA..I.IY..LE.GYVQRDTPRFV.N.G

C.hentzi --------------------------SNYKK.PLL..D.R.MQKE..L.VAYKMNKNQHENIVK.E.N.T.HVA..I.IY..LE.GYVQRDTPRFV.N.G

C.noxius --------------------------FNYKK.PLL..D.Q..QKE..L.VAYKMNKN.HENIVK.E.N.T.HVA..I.IY..LE.GYVQRDTPRFV.N.G

C.limpidus --------------------------FNYKK.PLL..D.Q..QKE..L.VAYKMNKN.HENIVK.E.N.T.HVA..I.IY..LE.GYVQRDTPRFV.N.G

C.orizaba --------------------------FNYKK.PLL..D.Q..QKE..L.VAYKMNKN.HENIVK.E.N.T.HVA..I.IY..LE.GYVQRDTPRFV.N.G

C.ochraceus --------------------------LDYKK.PLL..D.Q..QKE..L.VAYKMNKN.HENIVK.E.N.T.HVA..I.IY..LE.GYVQRDTPRFV.N.G

C.hirsutipalpus --------------------------YNYKK.PLL..D.Q..QKE..L.VAYKMNKN.HENIVK.E.N.T.HVA..I.IY..LE.GYVQRDTPRFV.N.G

T.trivittatus ----------------------------LRK.EML..N.Q.LHNE..L.T.FKMNKR.HEY.VK.E.N.T.QIA..I.IY..IE.GLVQRDTPRIV.N.G

L.abdullahbayrami ----------------------------IRKFSML..D.Q.RQKE..L.T.FKMNKR.HEYIVK.E.N.T.HVA..I.IY..IE.GYVQRDTPRIV.N.G

M.martensii ----------------------------IRKFNML..D.Q.LQKES.L.T.FKMNKRQHEY.VK.E.N.T.HVA..I.IY..IQ.GYVQRDTPRIV.N.G

H.aztecus ----------------------------.KR.SML..N.Q.TQPE..L.T.YKMNKRDYEYIVK.E.N.T.HVA..I.IY..EE.GYVERDTPRVV.E.G

H.concolorus ----------------------------.KR.SML..N.Q.TQPE..L.T.YKMNKRDYEYIVK.E.N.T.HVA..I.IY..EE.GYLERDTPRVV.E.G

S.donensis ----------------------------QKR.NML..N.Q.TQPE..L.T.FKMNKKDYEYIVK.E.N.T.HVA..I.IY..KE.GYVERDTPRAV.E.G

S.gertschi ----------------------------.KK.SML..N.Q.TQPE..L.T.F.MNKSDHEYIVK.E.N.T.HVA..I.IY..KE.GYMERDTPRAV.E.G

C.coahuilae ----------------------------.KK.SML..N.Q.TQPE..L.T.F.MNKSDHEYIVK.E.N.T.HVA..I.IY..KE.GYMERDTPRAV.E.G

P.schwenkmeyeri ----------------------------.KK.SML..N.Q.TQPE..L.T.F.MNKSDHEYIVK.E.N.T.HVA..I.IY..KE.GYMERDTPRAV.E.G

T.cristimanus ----------------------------.KK.SML..K.Q.TQPE..L.T.F.MNKSDHEYIVK.E.N.T.HVA..I.IY..KE.GYMERDTPRAV.E.G

M.gertschi ----------------------------.KT.SML..N.H.TQPE..L.TAFKMNKRDHEYIVA.E.N.T.HVA..I.IY..TE.GYVERDTPRAI.E.G

A.pococki_bajae ----------------------------.SR.NML..N.Q.TQPE..L.T.FKMNKRNHEYIVK.E.N.T.HVA..I.IY..EKSGYVERDTPRVV.E.G

D.melici ----------------------------.RT.SML..D.Q.TQPE..L.T.FKMNRHDYEYIVR.Q.N.T.HVA..I.IY..EE.GYVERDTPRVV.E.G

U.yaschenkoi ----------------------------.KT.NML..N.Q.TQPE..L.TAFKMNKR.HEYIVK.Q.N.T.HVA..I.IY..KE.GYVERDTPRAV.E.G

P.imperator ----------------------------.RM.NML..D.Q.TQPE..L.T.FKMNRNGHEYIVK.Q.N.T.HVA..I.IY..KE.GYVERDTPRAV.E.G

**110 120 130 140 150 160 170 180 190 200**

**....|....|....|....|....|....|....|....|....|....|....|....|....|....|....|....|....|....|....|....|**

**R.norvegicus** ---------DEGT-CT--DKANILYAWARNAPPTRLPKGVGFRVGGETGSKYFVLQVHYGDISAFRDNHKDCSGVSVHL---TRVPQPLIAGMYLMMSVD

*phm*-PAM

PHM*m*

C.sculpturatus ---------IHSAL.---K.MT.M....K....IT..PD...H...NSSIH.II..L..A--NPLPEGAS.N..ITL.M---..KR.KY...IH.LL.GS

C.hentzi ---------IHSAL.---K.MT.M....K...SIT..PD...H...NSSIH.II..L..A--NPLPEGAS.N..ITL.M---..KR.KY...IH.LL.GS

C.noxius ---------IHSAL.---K.MT.M....K....IT..PD...H...NSSIH.II..L..A--NPLPEGTS.N..ITL.M---..KR.KY...IH.LL.GS

C.limpidus ---------IHSAL.---K.MT.M....K....IT..PD...H...NSSIH.II..L..A--NPLPEGAS.N..ITL.M---..KR.KY...IH.LL.GS

C.orizaba ---------IHSAL.---K.MT.M....K....IT..PD...H...NSSIH.II..L..A--NPLPEGAS.N..ITL.M---..KR.KY...IH.LL.GS

C.ochraceus ---------IHSAL.---K.MT.M....K....IT..PD...H...NSSIH.II..L..A--NPLPEGAS.N..ITL.M---..KR.KY...IH.LL.GS

T.trivittatus ---------IHS.L.---K.MT.M....K....VT..PD...H..RNSSIR.VI..L..A--NLLPEGVS.D..ITL.M---..KR.KY...IH.LL.GS

L.abdullahbayrami ---------MHS.L.---K.MTVM...GK...TLT..PD...H...NSSVN.II..L..A--NSLPEGET.S...TL.M---..KR.KY...IH.LL.GS

M.martensii ---------MHS.L.---K.MTVM...GK...MLT..PD...H...NSSIN.II..L..A--NPLPEGET.S...TL.M---..KH.KY...IH.LL.GS

H.aztecus ---------IHSKL.---KGMT.M....K....VT..PD...H.S.NSSIR.VI..L..A--KPLPE.EI.R..LML.M---..IQ.KY...IH.LLASR

H.concolorus ---------IHSKL.---KGMT.M....K....VT..PD...H.S.NSSIR.VI..L..A--KPLPE.EI.R..LML.M---..IQ.KY...IH.LLASR

M.gertschi ---------IHSKL.---KGMT.M....K....VT..PD...H.S.NSSIR.VI..L..A--KPLPE.EI.R..LTL.I---...Q.KY...IH.LLASN

U.yaschenkoi ---------IHSKL.---KGMT.M....K....VT..PD...H.S.NSSIR.II..L..A--KPLTE.EV.R..LML.M---...Q.KY...IH.LLASN

P.imperator ---------IHSRL.---KGMT.M....K....VT..PD...H.S.NSSIR.II..L..A--KPLTG.EV.R..LML.M---..IQ.KY...IH.LLASR

T.atrox* ---------IHSKL.---KGMT.M....K....VT..SD...H.S.NSSIR.VI..L..A--KPLPE.EN.R..LML.M---..IQ.KY...IH.LLAGN

C.coahuilae* ---------IHSKL.---KGMT.M....K....VT..SD...H.S.NSSIR.VI..L..A--KPLPE.EN.R..LML.M---..IQ.KY...IH.LLAGN

D.melici* ---------IHSKL.---KGMT.I....K..S.VT..QD...H.S.NSSIR.II..L..A--KPLTG.EV.R..LMLYM---..------------LANN

**D.melanogaster** EMNRASQEESASP-.GPHSNSQ.V.....D.QKLN..E....K..KNSPI..L......AH.DK.K.GST.D...FLDY---.EE.RKKL..TL.-LGT.

C.sculpturatus EMAGSKSEYVSAPT.A--SGSQVI.....D...LD..D....K..KGS.IN.L......A.VTR.INGGT.N..IIIT.LPG.DPSVTKR..V..-LGTG

C.hentzi EMAGSKSEYISAPT.A--SGSQVI.....D...LN..D....K..KGS.IN.L......A.VTR.INGGT.N..IIIT.LPG.DPSVTKR..V..-LGTS

C.noxius EMAGSKSEYVSAPT.A--SGSQVI.....D...LD..D....K..KGS.IN.L......A.VTR.INGGT.N..IIIT.LPG.DPSVTKR..V..-LGTG

C.limpidus EMAGSKSEYVSAPT.A--SGSQVI.....D...LD..D....K..KGS.IN.L......A.VTR.INGGT.N..IIIT.LPGSDPSVTKR..V..-LGTG

C.orizaba EMAGSKSEYVSAPT.A--SGSQVI.....D...LN..D....K..KGS.IN.L......A.VTR.INGGT.N..IIIT.LPA.DPSVTKR..V..-LGTG

C.ochraceus EMAGSKSEYVPAPT.A--SGSQVI.....D...LE..D....K..KGS.IN.L......A.VTR.INGGT.N..IIIT.LPG.DPSVTKR..V..-LGTS

C.hirsutipalpus EMAGSKSEYVSAPT.A--SGSQVI.....D...LD..D....K..KGS.IN.L......A.VTR.INGGT.N..IIIT.LPG.DPSVTKR..V..-LGTG

T.trivittatus EMIGSKSEYVSAPI.A--QGSQVI.....D...LN..Q....K..KGS.IN.L......A.VTR.LNGGT.N..IILT.LPSSDSSVTKR..V..-LGTG

L.abdullahbayrami EMAGSLSEYKS.PT.A--EGSQ.I.....D...LE.......K..KGS.IN.L......A.VTK.INGGT.N..IVLT.LPGNDKSVTKR..V..-LGTN

M.martensii EMAGSHSGYQSAPT.A--EGSQ.I.....D...LE.......K..KGS.IN.L......A.VTK.INGGT.N..IVLT.LPGDDQSVTKR..VH.-LGTN

H.aztecus EMAGLKSGYRRAPT.N--SGSQVI....KD..ALQ..E....K..KG.DV.FL......ASVKN.INGAT.N..IILTIKPG.DKSVSKR..V..-LGTA

H.concolorus EMAGLKSEYRRAPT.N--SGSQVI....KD..ALQ..E....K..KG.DV.FL......ASVKN.INGGT.N..IILTIKPGNDKSVSKR..V..-LGTA

S.donensis EMAGSKAEYHRAPT.A--EGSQVI....KD..ALQ..E....K..KG.DVNFL......ASVKN.INGGT.S..IIIT.KPSSDKGVSKR..V..-LGTS

S.gertschi EMAGSKSEYHRAPT.Q--SGSQVI....KD..ALQ..N....K..KH.DVLFL......ATVDN.INGGT.N..IVIT.KPS.DKSVSKR..V..-LGTS

C.coahuilae EMAGSKSEYHRAPT.E--SGSQVI....KD..ALQ..N....K..KH.EVLFL......ATVDS.ING.T.N..IIIT.KPS.DKSVSKR..V..-LGTS

P.schwenkmeyeri EMAGSKSEYHRAPT.E--SGSQVI....KD..ALQ..N....K..KH.EVLFL......ATVDS.ING.T.N..IIIT.KPS.DKSVSKR..V..-LGTA

T.cristimanus EMAGSKSEYHRAPT.E--SGSQVI....KD..ALQ..N....K..KH.EVLFL......ATVDS.ING.T.S..IIIT.KPS.DTSVSKR..V..-LGTS

M.gertschi EMSGSRAEFHRAPT.G--SGSQVI....KD..ALQ.......K..KG.DVNFL......ASVEN.INGGS.N..IIIT.KPS.DGGVTKR..V..-LGTS

A.pococki_bajae EMAGSKSDYHRAPT.E--SGSQVI....KD..ALQ..E....K..KG.DVSFL......ATVEN.INGGT.S..IIIT.KPS.DGSVTKR..V..-LGTS

D.melici EMAGSKSDYHRSGT.K--TGSQVI....KD..ALQ.......K..KG.DV..L......AS.EK.VNGGT.N..IIIT.KPSSDRSVTKR..V..-LGTG

U.yaschenkoi EMAGSESDFRRAGT.R--SGSQVV....KD..ALQ..E....K..KG.DVQ.L......ASVDS.INGGT.S..IIIT.KPSSDESVSKR..V..-LGTS

P.imperator EMAGSRSDFHRAAT.G--SGSQVV.....D..ALQ..E....K..KG.DVH.L......ASVEN.INGGT.N..IIIT.KPSSDKNVTKR..V..-LGTA

**210 220 230 240 250 260 270 280 290 300**

**....|....|....|....|....|....|....|....|....|....|....|....|....|....|....|....|....|....|....|....|**

**R.norvegicus** TVIPPGEKVVNADISCQYK-MYPMHVFAYRVHTHHLGKVVSGYR--VRN---GQWTLIGRQNPQLPQAFYPV-EHPVDVTFGDILAARCVFTGEGRTEAT

*phm*-PAM

C.sculpturatus AM...KSPKYHV.VN.YFRGRQ.I.T......A.KY.V.I...KYNIKD---NA..FLAKG...W..T...M-DQVYT.SE..V.....TYNSTQSN.PI

C.hentzi AM...KSPKYHV.VN.YFRGRQ.I.T......A.KY.V.I...KYSIKD---NA..FLAKG...W..T...M-DQVHT.SE..V.....TYNSTQSN.PI

C.noxius AM...KSPKYHV.VN.YFRGRQ.I.T......A.KY.V.I...KYSIKD---NA..FLAKG...W..T...M-DQVYT.SE..V.....TYNSTQSN.PI

C.limpidus AM...KSPKYHV.VN.YFRGRQ.I.T......A.KY.V.I...KYSIKD---NA..FLAKG...W..T...M-DQVYT.SE..V.....TYNSTQSN.PI

C.orizaba AM...KSPKYHV.VN.YFRGRQ.I.T......A.KY.V.I...KYSIKD---NA..FLAKG...W..T...M-DQVYT.SE..V.....TYNSTQSN.PI

C.ochraceus AM...KSPKYHV.VN.YFRGRQ.I.T......A.KY.V.I...KYSIKD---NA..FLAKG...W..T...M-DQVYT.SE..V.....TYNSTQSN.PI

T.trivittatus AM...KSPKHHV.VN.YFRGRQ.I.T......A.KF.V.IT..KYSIKE---NV..SLAKG...W..T...M-DQVYTISE..V.....TYNSTQSNIPI

L.abdullahbayrami S....HRPKYHV.AN.YFRSRV.I........A.KF.V.IT..KYDIK.---K...FLAKG...W..T.F.M-DQTYTISQ..V..V..TYNSTQSNVPI

M.martensii S....HRPKYHV.AN.YFRSRV.I........A.KF.V.IT..KYDIK.---K...FLAKG...W..T.F.M-DQTYTISQ..V..V..TYNSTQSNVPI

H.aztecus A....HRPKFHV.VN.YV.SSV.I.P......A.Q..V.I...HYNSK.---RS.SFL.KA..HW....F.M-GRIHT..TD.V..L..T.NSSL.STS.

H.concolorus A....HMPKFHV.VN.YV.SSV.I.P......A.Q..V.I...HYNSK.---RS.SFL.KA..HW....F.M-GRIHT..TD.V..L..T.NSSL.STS.

M.gertschi A....HRPKFHV.VN.YV.SSV.I.P......A.Q..V.I...QYNFK.---.S..FLAKG...W....F.M-D.IHT.AT..L..L..I.NSSL.SVS.

U.yaschenkoi AI...HKPKFHV.VN.YI.SSV.I.P......A.Q..V.I...QYSFK.---RS.IFLAKG...W......M-D.IHT..A..A..L..T.NSSL.SVS.

P.imperator A....HRPKFHV.VN.YV.SSV.I.P......A.Q..V.I...QYSSK.---RS.IFLAKG...W......M-D.IHT..E..A..L..T.NSSL.SVP.

T.atrox* AI...HKPKFHV.VN.YA.SSV.I.P......A.Q..V.I...KYNFK.---.S..FLAKG...W....F.M-D.IHT.VA..V..L..T.NSSL.STP.

C.coahuilae* AI...HKPKFHV.VN.YA.SSV.I.P......A.Q..V.I...KYSFK.---.S..FLAKG...W....F.M-D.IHT.IA..V..L..T.NSSL.STP.

D.melici* AF.L.HRPKFHV.VN.YM.SSV.I.P......A.Q..V.I...QYSTK.---NS.IFLAKG...W......M-DYIHT..A..V..S..TYNSSL.SVS.

**D.melanogaster** GQ..AMKTE-HLETA.EVNEQKVL.P........G.........VRTNSDGEQE.LQL.KRD.LT..M..NTSNTDPIIE-..KI.V..TMQS-T.HRT.

PHM*m*

C.sculpturatus GYAR.ESEE-RFETA.KIDKPLIL.P..F.T...A........VIKGKD---.S.K...KH..LE..M....EDKNLIIEQ..V.....TMYN-F.DRI.

C.hentzi GFAL.KSEE-RFETA.KIDKPLTL.P..F.T...A........VIKGKD---.R.K...KHD.LE..M....EDKSLI.EE..V.....TMYN-F.DRV.

C.noxius GFAR.NSTE-RFETA.KIDKPLII.P..F.T...A........VIKGKD---.S.K...KH..LE..M....EDKNLIIEQ..V.....TMYN-F.DRI.

C.limpidus GFAL.KSEE-RFETA.KIDKPLIL.P..F.T...A........VIKRKD---.S.K...KH..LE..M....EDKNIIIEQ..V.....TMYN-F.DRI.

C.orizaba GFAE.KSEE-RFETA.RIDKPLIL.P..F.T...A........VIKEKD---.N.K...KHD.LE..M....EDKTLTIEQ..V.....TMFN-F.DRI.

C.ochraceus GFAL.KSEE-RFETA.KIDKPLIL.P..F.T...A........VIKGKD---.S.K...KHD.LE..M....EDKNLIIEQ..V.....TMYN-F.DRI.

C.hirsutipalpus GFARAKSEE-RFETA.KIDKPLIL.P..F.T...A........VIKEKD---.S.K...KH..LE..M....KDKSLIIEQ..V.....TMFN-F.DRI.

T.trivittatus GG.HAKQQE-HMETA.KIDKPIVL.P..F.T...A........VIERDS---.K.K...KHD.LE..M....EDESLI.QQ........TMYN-F.NRV.

L.abdullahbayrami G...AHKT.-PMETA.KIEESLTL.P..F.T...A........VIKAN.---KE.K...KHD.LK..M....ENQKLVIEK........TMYN-F.NRM.

M.martensii GA..AHKI.-HMETA.KIEQPVTL.P..F.T...A........VIKAN.---KR.K...KHD.LE..M....ENQDLTIEK........TMHN-F.NRI.

H.aztecus GE...KKTE-YMETA.TINEPLVL.P..F.T...V..E..A..VKKGK-----R.KR..KH...E..M....EDKTLTIKHN.VV....TMYN-F.NRI.

H.concolorus GE...RKTE-YMETA.TINEPLVI.P..F.T...V..E..A..VKKGK-----R.KR..KH...E..M....EDKTLTIKHN.VV....TMYN-F.NRI.

S.donensis GE.Q.K.TE-YMETA.TINEPLLI.P..F.T...A..E..AA.VKKG.-----E..R..KH...E..M....DDKRLTIKQ..VV....TMYN-F.KRV.

S.gertschi GE...K.TE-YMETA.TVNEPLEI.P..F.T...A..E..A..VKKGK-----I..R..KH...E..M....DDKRLIIEQ..V.....TMYN-F.G.T.

C.coahuilae GR.E.K.TE-YMETA.TINEPLVI.P..F.T...A..E..A..VIKE.-----K..R..KH...E..M....DDKRLVIRQN.VV....TMYN-F.K.T.

P.schwenkmeyeri GR.E.K.TE-YMETA.TINEPLVL.P..F.T...A..E..A..VIKEK-----K..R..KH...E..M....DDKRLVIREN.VV....TMYN-F.K.R.

T.cristimanus GR.KA..RE-YMETA.TINEPLVI.P..F.T...A.....A..VIKEG-----M.IR..KHD..E..M....DNKRLVIRQN.VV....TMYN-F.K.T.

M.gertschi GS...KQTE-YMETA.TITEPLVL.P..F.T...A...I.T..VKKGY-----R.K...KH...E..M....EDKSLKIRP..VV....TMYN-F.KRV.

A.pococki_bajae GA..AK.TE-YMETA.TINEPLVI.P..F.T...A.....A..VLKGK-----R.IR..KH...E..M...IDDKSLTIKQF.TV....TMYN-Y.KRV.

D.melici GFV.AKGTE-HMETA.KM.EPLVI.P..F.T...A.....A..VKKGN-----S..R..KH...E..M....DDKSLTIEK..VV....TMYN-F.ARI.

U.yaschenkoi GF.Q.R.TE-YMESA.TINEPVVI.P..F.T...A.....A..VKKGN-----T..R..KHD..K..M....DDRTLIIER..TV....TMYN-F.NRV.

P.imperator GS.R.R.TE-YMETA.TI.EPLVI.P..F.T...I...A.A..VKQGD-----L..R..KH...E..M....DDKSLTIQKD.VV....TMYN-F.NRV.

**310 320 330 340 350 360 370 380 390**

**....|....|....|....|....|....|....|....|....|....|....|....|....|....|....|....|....|....|....|...**

*phm*-PAM

**R.norvegicus** **HIGGTSSDEMCNLYIMYYMEAKYALSFMTCTKNVAPDMFRTIPAEANIPIPV**KPDMVMMH-GH-HKEAE--NKEKSALMQQPKQGEEEVLEQ------

C.sculpturatus YM.S.AN...........TN.NDGS..AR.LDIEI.KLVNEL..DSDV.LSPN.ALEENA----.TN-Q--------WN.KTSVS-------------

C.hentzi YM.S.AN...........TN.NDGS..AR.LDIEI.KLVNEL..DSDV.L.SN.ALEENA----.TN-Q--------WN.KTSVS-------------

C.noxius YM.S.AN...........TN.NDGS..AR.LDIEI.KLVNEL..DSDV.L.PN.ALEENS----.TS-Q--------WN.KTAVS-------------

C.limpidus YM.S.AN...........TN.NDGS..AR.LDIEI.KLVNEL..DSDV.L.PN.ALEENA----YTN-Q--------WN.KTSVS-------------

C.orizaba YM.S.AN...........TN.NNGS..AR.LDIEI.KLVNEL..DSDV.L.PN.ALEENA----.TN-Q--------WN.KTSIS-------------

C.ochraceus YM.S.AN...........TN.NDGS..AR.LDIEI.KLVNEL..DSDV.L.PN.ALEENA----.TN-Q--------WN.KTSVS-------------

T.trivittatus YM.S..N...........TS.DEGS..AR.LDVEI.KLVNEL.PDSD..L.PNSELEENA----.TS-Q--------WN.K.SIS-------------

L.abdullahbayrami YM.S.AN.......L...TN.NGGNK.AR.LDVEI.KLVNEL.TGSDV.L.PN.A.EEEA----.NNHQ--------WN.ISAMS-------------

M.martensii YM.S.AN.......L...TN.NGGNK.AR.LDVEI.KLVNEL.SGSDV.L.PN.A.EEEA----.ASHQ--------WN.ISSMS-------------

H.aztecus YM.S..N...........TR.NRGY..DR.IDVEV.QLVNML.SGND..L.RN.A.EEHAQ..P.DDEQHPADYINVWS.K.PVY-------------

H.concolorus YM.S..N...........TR.NRGH..DR.IDVEV.ELVNML.SGTD..L.RN.A.EEYAQ..P.DDEQHPADYINVWS.K.PVY-------------

M.gertschi YM.SA.............TR.DGGH..DR.IDVEV.HLVSML.NGNDV.L.RN.A.EEHAQ..P.DNEQYYSVY.NEWN.K.SVH-------------

U.yaschenkoi YM.S..N...........TR.DGGH..GR.IDIEV.QLVKML.NGNDV.L.RN.VLEEHAQ..L.DDEQYSASYGN.WS.K.SVY-------------

P.imperator NM.S.TN...........TR.DGGH..DR.VDIEV.QLIKML.NGNDV.L.RN.SLEEHAQ..L.NDEQYSEIYGNSWS.K.SVY-------------

T.atrox* YM.S..N...........TR.DRGR..DR.IDVEV.---------------------EHAQ..L.D.EHYSAAYGNSWD.K.SVH-------------

C.coahuilae* YM.S..N...........TR.DRGR..DR.IDVEV.QLVNML.SGND..L.RN.A.EEHAQ..L.D.EHYSAAYGNSWD.K.----------------

D.melici* YM.S..D...........TRSDGGH..GR.VDIEV..LA.ML.SGNDV.L.RN.ALEEDAQ..L.D-EQYFSTYGNTWN.K.SVY-------------

**D.melanogaster** K..P.NE.....F.L...VDHGET.NMKF.FSQG..YY.WSN.---DSGLHNI.-------------------------HIEAST----.--------

C.sculpturatus SV.A.AE.....F.M...VNGNDI.DDKY.FSMGP.GYYWKN----D.L.DKI.E----------------------WVEEDASS----.RK------

C.hentzi SV.A.AE.....F.M...VNGNDI.DEKY.FSMGP.GYYWKN----DLL.GKI.E----------------------WVEEDASS----.RK------

C.noxius SV.A.AE.....F.M...VNGNDI.DDKY.FSMGP.GYYWKN----DLL.DEI.E----------------------WVEKDASS----.RK------

C.limpidus SV.A.AE.....F.M...VNGNDI.DEKY.FSMGPSGYYWKN----D.L.DKI.E----------------------WVEEDASS----.RK------

C.orizaba SV.A.AE.....F.....VNG.DI.DDKY.FSMGP.GYYWDN----D.L.NKI.E----------------------EVDKNASS----.RK------

C.ochraceus SV.A.AE.....F.M...VNGNDI.DEKY.FSMGP.GYYWKN----D.L.GEV.K----------------------WVEEDASS----.RK------

C.hirsutipalpus SV.A.AE.....F.M...VNGNDI.DDKY.FSMGP.GYYWKN----D.L.DKI.E----------------------WVEDGASS----.RK------

T.trivittatus L..L.AE.....F.M...V.GDEI.DDKY.FSMGP.AYYWEN----DEL.GDI.K----------------------WVDKDAST----..K------

L.abdullahbayrami F....AD.....F.M...VNGDKI.DDRY.FSDGP.AY.WEN----DSQ.KNV.S----------------------WVDEDAST----.DR------

PHM*m*

M.martensii F....AD.....F.M...VNGDEI.NDKY.FSDGP.AY.WET----DSQ.KNV.L----------------------WVDEDAST----.DR------

H.aztecus YV.A.AE.....F.....TNGDQI.DQKY.FSAGP.NYYWDT----DPQ.GNP.G----------------------WINRDASS----.YK------

H.concolorus YV.A.AE.....F.....TNGDQI.DQKY.FSAGP.NYYWDT----DPQ.GNP.A----------------------WINRDASS----.YK------

S.donensis Y..S.AE.....F.M...TNGDQI.DDKY.FSAGP.YYYWET----DPQVGKP.R----------------------WVNRDAST----.DK------

S.gertschi Y..A.AK.....F.M...T.GDQI.KEKY.FSAGP.YYYWDT----DPQVGKP.R----------------------WVNKDAST----.DE------

C.coahuilae Y..A.AK.....F.M...T.GDRI.NDKY.FSAGP.FYYWNT----DPQVGKP.R----------------------WVNEDAST----.FE------

P.schwenkmeyeri Y..A.AK.....F.M...T.GDRI.NDKY.FSAGP.FYYWDT----DPQVGKP.R----------------------WANKDAST----.DE------

T.cristimanus FV.A.AK.....F.M...T.GDRI.NDKY.FSAGP.FYYWNT----DPQVGEL.R----------------------SVNEDASR----.YE------

M.gertschi NV.A.AN.....F.V...TDGDRI.DEKY.FSQGP.YYYWET----DPQLGKV.R----------------------.INKDAST----.DK------

A.pococki_bajae YV.A.AK.....F.M...TDGDRI.DDKY.FSSGP.YYYWET----DPQVGSP.R----------------------WVNKDAST----.DK------

D.melici YV.P.GD.....F.M...VDGDKI.DDKY.FSSGP.YYYWDD----DPKVGRMAG----------------------WVNKDAST----IRNR-FSQS

U.yaschenkoi SV.P.AE.....F.M...VDGDKI.DSKY.FSAGP.YYYWET----DPQVGKP.R----------------------WVNKDAST----I--------

P.imperator YV.P.AE.....F.M...VNGDKI.DDKY.FSAGP.YYYWET----DPQVGKQ.R----------------------WVNKDAST----IKKRNFKY-

**Supplementary Figure S5.** Sequence alignment of PHM domains. Sequences of the scorpion *phm*-PAM and PHM*m* domains are shown aligned with sequences of *phm*-PAM2 from *Rattus norvegicus* (Uniprot:P14925) and PHM from *Drosophila melanogaster* (Uniprot:O01404). Color codes are as follows: residues involved in copper coordination CuH (H^107^, H^108^ and H^172^) and CuM (H^242^, H^244^ and M^314^); cysteine residues involved in disulfide bond formation (C); residues relevant to catalytic activity (R^240^, N^316^ e Y^318^); sequence not determined (--). The residue numbering of the PAM1 isoform from *R. norvegicus* is used*.*
