# Supplementary material for: The Dual α-Amidation System in Scorpion Venom Glands
Source: Toxins (Basel). 2019 Jul 20;11(7):425. doi: 10.3390/toxins11070425 (PMC6669573; doi:10.3390/toxins11070425)
Supplement: Supplementary file 1 [file toxins-11-00425-s001.zip › Delgado-Prudencio Supplementary_Figure_S6_R2 v4.docx]

**10 20 30 40 50 60 70 80 90 100**

**....|....|....|....|....|....|....|....|....|....|....|....|....|....|....|....|....|....|....|....|**

**R.norvegicus**  -----------------DF---------------------HVEE--------------------------------------------ELDWPGVYLLPG

*pal*-PAM

PAL*m*

C.sculpturatus -----------------E.---------------------TYK.--------------------------------------------VAS..AEDRKF.

C.hentzi -----------------E.---------------------TYK.--------------------------------------------VAS..AGDRKF.

C.noxius -----------------E.---------------------TYK.--------------------------------------------VAS..AEDRKF.

C.limpidus -----------------E.---------------------TYK.--------------------------------------------VAS..AEDRKF.

C.orizaba -----------------E.---------------------TYK.--------------------------------------------IAS..AEDRKF.

C.ochraceus -----------------E.---------------------TYK.--------------------------------------------VAS..AEDRKF.

T.trivittatus -----------------..---------------------TYK.--------------------------------------------IAS..TEKGKF.

L.abdullahbayrami -----------------..---------------------TYK.--------------------------------------------VAS..NEGERF.

M.martensii -----------------..---------------------TYK.--------------------------------------------VAS..NEGEKF.

H.aztecus -----------------..---------------------KYK.--------------------------------------------VEN..TEKTKY.

H.concolorus -----------------..---------------------KYK.--------------------------------------------VEN..TEKPKY.

M.gertschi -----------------..---------------------KYK.--------------------------------------------VEN..DEITKY.

U.yaschenkoi -----------------..---------------------KYK.--------------------------------------------.EN..TENMKF.

P.imperator -----------------..---------------------KYK.--------------------------------------------.EN..TENIKF.

D.melici -----------------..---------------------KYK.--------------------------------------------.EN..TEKIKF.

T.cristimanus -----------------..---------------------KYK.--------------------------------------------VEN..TEKTKY.

C.coahuilae* ---------------------------------------------------------------------------------------------TEKTKY.

**D.melanogaster** -----SSSNHLPAGLAM.LGPGVNLNERFFD-QVRALIKRRLQ.KGLAKPEQPELAMPLTDDDAVALQNQRSYDNVPLPAASVPTPVLVEN..TEQHSF.

C.sculpturatus AGFYPEPAEYFYPEQ---.-LDYKTNDKFLR---NENTRISI..--------------------------------------------QVE..PENIFRD

C.hentzi AGFYPEPAEYFYPEQ---.-LDYRTNDKFLR---TENTRIS...--------------------------------------------QVE..PENIFKD

C.noxius AGFYPEPAEYFYPEQ---.-LDYKTNDKFLR---NENTRISI..--------------------------------------------QVE..PENIFKD

C.limpidus AGFYPEPAEYFYPEQ---.-LDYKTNDKFLR---NENTRISI..--------------------------------------------QVE..PENIFKD

C.orizaba AGFYPEPAEYFYPEQ---.-LDYKTNDKFLR---NENTRISI..--------------------------------------------QVE..PENIFKD

C.ochraceus AGFYPEPAEYFYPEQ---.-LDYKTNDKFLR---TENTRISI..--------------------------------------------QVE..PENIFKD

C.hirsutipalpus AGFYPEPAEYFYPEQ---.-LDYKTNDKFLR---NDNTRISI..--------------------------------------------QVE..PENIFKD

T.trivittatus AGFYPEPAEYFYPEQ---.-LDYQTNDKPGG---NENRKMSI..---------------------------------------------VE..VENTFKN

L.abdullahbayrami -SFRGWPSR--FPSR-----GDYTQTNRFSGIRKTDNIRIS.R.--------------------------------------------.IN..DKST.KN

M.martensii -SFRGWPAR--YPSR-----EDYTQNNRFSGIRKTDNIRIS...--------------------------------------------.IN..HKST.KN

H.aztecus -RSHYEADKYWYDKLQ-GL-ESRK-NNRFLA---SKNEDF-IR.--------------------------------------------VIG..DNDV.N.

H.concolorus -RSHYEADKYWYDKLQ-GL-ESRRENNRFLA---SKNEDF-IQ.--------------------------------------------VIG..DNDVFS.

M.gertschi -RSHYEADKYWFDKLQ-.L-ELRK-NNKFFG---SQNERI-IQ.--------------------------------------------VIG..YNDI.NS

C.coahuilae -RSHYEADKYWYDKLQ-GL-ESRRESNRFLG---SRSEGF-MQ.--------------------------------------------VIG..DTGS.KN

T.cristimanus -RSHYEADKYWYDKLQ-GL-ESRRESNRFLG---SRSEGL-IQ.--------------------------------------------VIG..DTDS.KN

P.schwenkmeyeri -RSHYEADKYWYDKLQ-GL-ESRRESNRFLG---SRSEGF-.Q.--------------------------------------------VIG..DTDS.KN

S.gertschi -RSHYEADKYWYDKLQ-GL-ESRRESNSLLG---SRSNGF-.Q.--------------------------------------------VT...DGDI.SD

A.pococki bajae -RSQYEADKYWYDKLQ-SL-EARKENSKFHG---SQKEGF-IQ.--------------------------------------------II....NDI.S.

D.melici -RSRFEADKYWYDKLQ-SL-ESKRENNKLFS---LQNKPA-IR.--------------------------------------------VI..ANSDVFN.

U.yaschenkoi -RSRYEADKYWYDQLQ-SL-ESKRQNNR-FS---SQNEPS-.Q.--------------------------------------------VIG.ADGDIFS.

**110 120 130 140 150 160 170 180 190 200**

**....|....|....|....|....|....|....|....|....|....|....|....|....|....|....|....|....|....|....|....|**

**R.norvegicus_** --QVSGVALDSKNNLVIFHRGDHVWDGNSFDSKFVYQQRGLGPIEEDTILVIDPNNAEILQSSGKNLFYLPHGLSIDTDGNYWVTDVALHQVFKLDPHSK

*pal*-PAM

PAL*m*

C.sculpturatus --..TA.DI..N..I..........N.L...VEDR.LLTEK...ASP..VTL.SVTGHV.HQW.S.I..M....TL.G.-YV.L....M..I..YPLSGD

C.hentzi --..TA.DI..N..I..........N.L...VEDR.LLTEK...ASP..VTL.SATGHV.HQW.S.I..M....TL.G.-YV.L....M..I..YPLSGD

C.noxius --..TA.DI..N..I..........N.L...VEDR.LLTEK...ASP..VTL.SVTGHV.HQW.S.I..M....TL.G.-YV.L....M..I..YPLSGD

C.limpidus --..TA.DI..N..I..........N.L...VEDR.LLTEK...ASP..VTL.SVTGHV.HQW.S.I..M....TL.G.-YV.L....M..I..YPLSGD

C.orizaba --..TA.DI..N..I..........N.L...VEDR.LLTEK...ASP..VTL.SVTGHV.HQW.S.I..M....TL.G.-YV.L....M..I..YPLSGD

C.ochraceus --..TA.DI..N..I..........N.L...VEDR.LLTEK...ASP..VTL.SATGHV.HQW.S.I..M....TL.G.-YV.L....M..I..YPLSGD

T.trivittatus --.IAA.DI..N.............N.L...VEDR.LLTEK...STP..VTL.SETGHVVHQW.S.I..M....TLYG.-YI.L....M..I..YSLLGD

L.abdullahbayrami --.ITA.DI.AN..I......N...N.L...VEDR.LLTEK...SSP..VTL.SATGH..HQW.S.I..M....TL.GN-YV.L.......I..YPLSGD

M.martensii --.IAA.DI.AN..I......N...N.L...VENR.LLTEK...SSP..VTL.SATGHV.HQW.S.I..M....TL.G.-YV.L.......I..YPLSGD

H.aztecus --..AA.DI.AQ.........N...NEQT..AENH..LTAN...SSP..VTL..ATGHL.NQW.S....M....TVHS.-YV.L....M.....YPLSGS

H.concolorus --..AA.DI.AQ.........N...NEQT..AENH..LTAN...SSP..VTL..ATGHL.NQW.S....M....TVHS.-YV.L....M.....YPLSGS

M.gertschi --..AA.DI.AQ..V......N...NEQT..AENH..LTEN...SSP.VVTL..ATGHL.SQW.S....M....T.QS.-YV.L..........FPLSGA

U.yaschenkoi --.IAA.DI.AQ.........N...NEQT..AENH..LTDR...SSP.VVTL..GTGHL.SQW.S....M....TVQR.-YM.L....M.....FPLSGS

P.imperator --..AA.DI.AQ.........N...NELT..AENR..LTNR...SSP..VTL..GTGHL.NQW.S....M....TVQN.-YV.L....M.....FPVSGE

D.melici --..AA.DI.AQ....V....N.I.NEQT..IENR..LIDR...SSP.VVTL..GTGHL.SQW.S....M....TVER.-YV.L....M.....FPLSGS

T.cristimanus --...A.DI.AQ.........S...NEQT..AENR..LTED...SSP.VVTV..ATGRL.NQW.S....M....TVQN.-YV.L....M.....FPLSGS

C.coahuilae* --...A.DI.AQ.........S...NEQT..AENR..LTED...SSP.VVTV..ATGRL.NQW.S....M....TVQN.-YV.L....M.....FPLSGS

**D.melanogaster** --..TA..V.PQGSP.V...AERY..V.T.NESNI.YLIEY...K.N..Y.L.AKTGA.KSGW.S.M..M....T..LH....I....M..A..FK.F.N

C.sculpturatus IR..A..DV.RLG.VHV.....RM..MR..K-GDN...QE....QI....IL..SSGQVIR.W...R.......TL.SHE.S............FASNNE

C.hentzi IR..A..DV.RLG.VHV.....RM..MR..K-GDN..LQE....QT....IL..SSGQVIR.W...R.......TL.SHE.S............FASNNE

C.noxius IR..A..DV.RLG.VHV.....RM..MR..K-GDN...QE....QI....IL..SSGQVIR.W...R.......TL.SHE.S............FASNNE

C.limpidus IR..A..DV.RLG.VHV.....RM..MR..K-GDS...QE....QI....IL..SSGQVIR.W...R.......TL.SHE.S............FASNNE

C.orizaba IR..A..DV.RLG.VHV.....RM..MR..K-GDN...QE....QI....IL..SSGQVIR.W...R.......TL.SHE.S............FASNNE

C.ochraceus IR..A..DV.RLG.VHL.....RM..MR..K-GDN...QE....QI....IL..SSGQVIR.W...R.......TL.SHE.S............FASNNE

C.hirsutipalpus IR..A..DV.RLG.VHV.....RM..MR..K-GDN...QE....QI....IL..SSGQVIR.W...R.......TL.SHE.S............FASNNE

T.trivittatus IL..A..EV.KWGQVHV.....RM..DR..K-GG...LEE.....T.AV.IL..SSG.LIRGW...R......ITL.NRE.S............FAVNNE

L.abdullahbayrami VQ....IDV.KLG.VHVL....RA..MW...-GNN..LQE....KS..V.ILNQSSG..IRKW...R.......T..N.E...I..........FT.NNE

M.martensii IR....IDV.KLG.VHVL....RA..MW...-GNN..L.E....KD..V.ILNQSSG..IRKW...R.......TV.N.E.S.I..........FT.NNE

H.aztecus IK..A...V.KSG.VH.....NR..NQL...FNNN...KN....AM...F.LNQTSG.VIK.W..RR..I...IT..N...S.L........M.FRDGD.

H.concolorus IK..A...V.KSG.VH.....NR..NQL...FNNN...KN....AM.....LNQTSGKVIK.W..RR..I...IT..N...S.L........M.FRDGD.

M.gertschi IK..A...V.ESG.VH.....NRE.NQF...YNGN.LEKN.....V.AV.ILNQHTGKVIR.W..RR.......T...N..S..........M.FRNGDE

C.coahuilae VK..A.I.V.KSG.VHV....NRE..Q.T..YNDN.L.KN.....VNAV..LNQYSGNVVR.W...R.......T..NN..T.I........M.FPDGDE

T.cristimanus VK..A.I.V.KSG.VHV....NRE..Q.T...NDN.L.KD.....V.A...LNQYSGNVVR.W...R.......T..NN..T.I........M.FPDGDE

P.schwenkmeyeri VK..A...V.KSG.VHV....NRE..Q.T..FNDN.L.KN.....VN....LNQHSGNVVR.W...R.......T..NN..T.I........M.FPDGDE

S.gertschi VK..A.I.V.KRG.VHV....NRE..Q.T..FNDN.L.KN.....VNA.I.LNQYSGNVVR.W...R.......T..NS..T..........M.FNNEDE

A.pococki bajae TK..A...V.KSG.VYV....NRE.NHL...YNDN.LEKN.....I.....LNQYSGKLMR.W..KR......IT..NN..S..........M.F.NGNE

D.melici IK..T...V..SG.IHV....NKE.N.L...LNDN.L..D....DI..VF.L.QHLGKL.R.W...R......ITV.SN..S..........M.FSAGDE

U.yaschenkoi IK..A...V..SGKVHV....NRE.N.L...VNNN.L.KD....DI.P.F.L.QHSG.LIR.W..KR......IT..SN..S.........IM.FSDSDE

**210 220 230 240 250 260 270 280 290 300**

**....|....|....|....|....|....|....|....|....|....|....|....|....|....|....|....|....|....|....|....|**

**R.norvegicus_** EGPLLILGRSMQPGSDQNHFCQPTDVAVEPSTGAVFVSDGYCNSRIVQFSPSGKFVTQWGEES--SGSSPRPGQFSVPHSLALVPHLDQLCVADRENGRI

*pal*-PAM

PAL*m*

C.sculpturatus IK.A.TF.ERFV..N.DK...K..S..IS-.N.DIY.A.........RL.S....LN...QSIQIGNFP.G..D.AI..KIT..EDKGLV..........

C.hentzi IK.V.TF.ERFV..N.DK...K..S..IS-.N.DIY.A.........RL.S....LN...QSIQIGNFP.G..D.AI..KIT.IEDKGLV..........

C.noxius IK.V.TF.ERFV..N.DK...K..S..IS-.N.DIY.A.........RL.S....LN...QSIQIGNFP.G..D.AI..KIT..EDKGLV..........

C.limpidus IK.V.TF.ERFV..N.DK...K..S..IS-.N.DIY.A.........RL.S....LN...QSIQIGNFP.G..D.AI..KIT..EDKGLV..........

C.orizaba IK.V.TF.ERFV..N.DK...K..S..IS-.N.DIY.A.........RL.S....LN...QSIQIGNFP.G..D.AI..KIT..EDKGLV..........

C.ochraceus IK.V.TF.ERFV..N.DK...K..S..IS-.N.DIY.A.........RL.S....LN...QSIQIGNFP.G..D.AI..KIT..EDKGLV..........

T.trivittatus IK.I.TV.ERFV..N.DK...K..S..IS-.N.DIY.A.........RLTS..M.LN...QSNEIGI.A.E..F.AI..KVT.IEDKGLV..........

L.abdullahbayrami IK.V.TV.ERFI....DK...K..S...S-.N.DIY.A........MRL.S..E.LN...QSNQIGV...A..FLDI..KIV.AEDKGLV.......R..

M.martensii IK.V.TV.ERFI..N.KK...K..S...S-TNADIY.A........IRL.S..E.LN...QSNQIGV...A..F.DI..KIV.AEDKGLV..........

H.aztecus IVSV.T..ERFT....HK...K..S...S-.N.DIY.A.........R..AE.D.MN...QSNPIGIN..PK.S..I..KVV.AEDKKLV.A........

H.concolorus IVSV.T..ERFT....HK...K..S...S-.N.DIY.A.........R..AE.D.MN...QSNPIGIN..PK.S..I..KVV.AEDKKLV.A........

M.gertschi ILSV.T..ERFI..N.HK...K..S..IS-.N.NIY.A.........R..SE.D.MN...QSNPFGVNF.PK.A.AI..KVI.AEDKQSV..........

U.yaschenkoi IVSA.T..ERFT....HK...K..S..LS-.N.DIY.A.........R..AE.D.MN...QSNPIGIN..PK.S.AI..KVV.AEDKQLV..........

P.imperator IV.A.T..ERFT....HK...K.AS..IS-.S.DIY.A.........R..SE.D.MN...QSNPIGVN..PK.S.AI..KVV.AEDKQLV..........

D.melici IVSA.T..EKFT....HK...K..S..IC-.S.DIY.A.........R..SE.E.LN...QI.PLGI...PE.S.AI..KVV.AEDKQLV.........V

T.cristimanus IMSV.T..ERFT....HK...K..S..IS-.N.NIY.A.........R..AE.N.MN...QSNPIGR...PK.S.DI..KVI.AEDKQLV.A........

C.coahuilae* IMSV.T..ERFT....HK...K..S..IS-.N.NIY.A.........R..AE.N.MN...QSNPIGR...PK.S.DI..KVI.AEDKQLV.A........

**D.melanogaster** -K...TI.KRFR...SVK.L.K..SI..A-T..EF.IA........LK.NAA..LLRTIPQ-------P.EFLSLQ...AIT.LE...L..I.....M.V

C.sculpturatus -E...V..QKFK....RS...K......S-.S.I............IA.K.D.T.HGEF.QQD----------KMN.....S.IEERKC........A..

C.hentzi -E...V..QKFK....RS...K......S-.S.I............IA.K.D.T.HGEF.QQD----------KMN.....S.IEERKC........A..

C.noxius -E...V..QKFK....RS...K......S-.S.I............IA.K.D.T.HGEF.QQD----------KMN.....S.IEERKC........A..

C.limpidus -E...V..QKFK....RS...K......S-.S.I............IA.K.D.T.HGEF.QQD----------KMN.....S.IEERKC........A..

C.orizaba -E...V..QKFK....RS...K......S-.S.I............IA.K.D.T.HGEF.QQD----------KMN.....S.IEERKC........A..

C.ochraceus -E...V..EKFK....RS...K......S-.S.I............IA.K.D.T.HGEF.QQD----------KM......S.IEERKC........A..

C.hirsutipalpus -E...V..QKFK....RS...K......S-.S.I............IA.KSD.T.HGEF.QQD----------KMN.....S.IEERKC........A..

T.trivittatus -E.SIV..KKF.....RE...K......A-.S.V.Y..........IA.Q.D.T.FGEFAQSD----------KMNI....S.IEERKL......Q.A..

L.abdullahbayrami -K.SIV..EEFT....RM...K.....IA-.S.YI.I.........IA.K.N.I.YGEF.KHE----------GM.I....SMIEEKKS........A..

M.martensii -K.SIV..EEFT....RT...K......T-.S.YI.I.........IA.K.N.T.YREF.KNE----------KMII....SMIEEKKC........A..

H.aztecus -L.S.V...EFEH...NE...K......L-.S.YF...........MM.R.D.H.HKKI.V.N----------NMFI....S.MEERSM........A..

H.concolorus -L.S.V...EFEH...NE...K......L-.S.YF...........MM.R.D.H.HKKI.V.N----------NMFI....SFMEERSM........A..

M.gertschi -L.S.V..KEFEH...NE...K......L-.S.YF...........MM.R.D.S.HKKI.A.D----------NLVI....S.MEERSM........A..

C.coahuilae -L.S....KEFEH...NE...K......L-.S.YF...........MM.K.D.S.HKKI.A.D----------KML.....SIMEERQM.......HA..

T.cristimanus -L.S....KEFKH...NE...K......L-.S.YF...........MM.K.D.S.HKKI.A.D----------HML.....SIMEERQT.......HA..

P.schwenkmeyeri -L.S....KEFEH...NE...K......L-.S.YF...........MM.K.D.S.HKKI.A.D----------KML.....S.MEERQM........A..

S.gertschi -S.S....KEFEH...NE...K......L-.S.YF...........MM.K.D.S.HKKI.A.D----------NML.....S.MEEKHM........A..

A.pococki bajae -L.S....KEFEH...NE...K......L-.S.YF...........MM.K.D.S.HKKI.TKD----------HMFI..G.S.MEERSM........A..

D.melici -L.S.V..KEFEH...RE...K......L-PS.YF...........MM.K.D.S.HKEI.A.D----------NML.......AKDRSK........I..

U.yaschenkoi -L.S.V..KEFEH...RE...K......L-LS.YF............M.K.D.S.HKEI.A.D----------HMF.....S..KEGSM.......A..

**310 320 330 340 350 360 370 380 390 400**

**....|....|....|....|....|....|....|....|....|....|....|....|....|....|....|....|....|....|....|....|**

**R.norvegicus_** QCFK--TDT----KEFVREIKHASFGRNVFAISYIP---GFLFAVNGKPYFGDQEPVQGFVMNFSSGEIIDVFKPVRKHFDMPHDIVASEDGT-VYIGDA

*pal*-PAM

PAL*m*

C.sculpturatus ...T--LMS----GN.HF..V.KE..GRL.S.D.S.SKG.LF...C.PSIYY-KH...A..F..T.RQLLNS...KQGT.TQ....AVTH...E.FVSEI

C.hentzi ...T--LMS----GN.HF..V.KE..GRL.S.D.S.SKG.LI...C.PSIYY-KH...A..F..T.RQLLNS...KQGT.TQ....AVTH...E.FVSEI

C.noxius ...T--LMS----GN.HF..V.KE..GRL.S.D.S.SKG.LF...C.PSIYY-KH...A..F..T.RQLLNS...KQGT.TQ....AVTH...E.FVSEI

C.limpidus ...T--LMS----GN.HF..V.KE..GRL.S.D.S.SKG.LF...C.PSIYY-KH...A..F..T.RQLLNS...KQGT.TQ....AVTH...E.FVSEI

C.orizaba ...T--LMS----GN.HF..V.KE..GRL.S.D.S.SKG.LF...C.PSIYY-KH...A..F..T.RQLLNS...KQGT.TQ....AVTH...E.FVSEI

C.ochraceus ...T--LMS----GN.HF..V.KE..GRL.S.D.S.SKG.LF...C.PSIYY-KH...A..F..T.RQLLNS...KQGT.TQ....AVTH...E.FVSEI

T.trivittatus ...T--LVS----GN.HF..I.EE..GRL.S.D.S.SKG.L....C.PSIHY-KH...A..F..T.RQLLNS...KQGT.TQ......TR...E.FVSEI

L.abdullahbayrami ...T--LM.----GN.HF..IRKE..EQL.S.D.T.IKG.L.Y..C.P-FRD-KH...A..F.LTNQQLLNS.T.KQGT.TQ....AVTH..SE.FVSEI

M.martensii ...T--LMS----GN.HF..IRKE..GRL.S.D.T.IKG.L.Y..C.PSFYN-KH...A..F.LTNQQLLNS.T.KQGT.TQ....AVTH..NE.FVSEI

H.aztecus ...T--LV.----GT.QFQ.T.KK..GRL.S.D.T.LRG.L.Y..C.PSEYF-DH...A.IF..TNQQLLNSLA.KQGS.TQ....TVTL..NE.FVSEI

H.concolorus ...T--LV.----GT.QFQ.T.KK..GRL.S.D.T.LRG.L.Y..C.PSEYF-DH...A.IF..TNQQLLNSLA.KQGS.TQ....TVTL..NE.FVSEI

M.gertschi ...T--LV.----GN.QFQ.TRKE..GRL.S.D.T.FKG.L.Y..C.PSEYF-DH...A..F..TNHQLLNSLA.KQGS.SQ....AITL..NE.FVSEI

U.yaschenkoi ...T--LI.----GN.RFQ.ARKE..GRL.S.D...IKG.L.Y..C.PSIYF-DH...A..F..TNQQLLNSLA.QQGS.TQ....TVTL..NE.FVSEI

P.imperator ...T--LI.----GN.QFQ.SRRE..GRL.S.D...IKG.L.Y..C.PSEYF-DH...A..F..TNQQLLNSLA.QQGS.TQ....AVTL..NE.FVSEI

D.melici ...T--LI.----GY.QFQ.A.KE..GRL.S.D.T.AKG.L.Y..C.PSLYVSNH..KA..F..T.RQLLNS.A.QQDN.TQ....TVTL..DE.FVSEI

T.cristimanus ...T--LV.----GN.QFQ.ARKE..GRL.S.D.TTTKG.L.Y..C.PSVYF-DH...A..F..T.QQLLNS.T.KQGS.TQ....AVTL..NE.FVSEI

C.coahuilae* ...T--LV.----GN.QFQ.ARKE..GRL.S.D.T.TKG.L.Y..C.PSVYF-DH...A..F..T.QQLLNS.T.KQGS.TQ....AVTL..NE.FVSEI

**D.melanogaster** V.P.AGLISSHGEG.PAAT.QEPDL.-R..GVASF---GDIV.....PTSML---..R..TIDPR.ET..GHW----GE.KN..SMAV.VN.SAL.VTEI

C.sculpturatus L.YS--LKE--DGSLGDLTVNNDVPTGAPY..VD---RGEYML..TLSYARE--GAAM.IT.KL.D.DV.NTWHSYEG-.S....MAITSN..FL.VV.V

C.hentzi L.YS--LKE--DGSLGDLTVNNDVPTGAPY..VD---RGEYM...TLSYARE--GAAM.IT.KL.D.DV.NTWHSYEG-.S....MAI.SN..FL.VV.V

C.noxius L.YS--LKE--DGSLGDLTVNNDVPTGAPY..VD---RGEYML..TLSYARE--GAAM.IT.KL.D.DV.NTWHSYEG-.S....MAITSN..FL.VV.V

C.limpidus L.YS--LKE--DGSLGDLTVNNDVPTGAPY..VD---RGEYML..TLSYARE--GAAM.IT.KL.D.DV.NTWHSYEG-.S....MAITSN..FL.VV.V

C.orizaba L.YS--LKE--DGSLGDLTVNNDVPTGAPY..VD---RGEYML..TLSYARE--GAAM.IT.KL.D.DV.NTWHSYEG-.S....MAI.SN..FL.VV.V

C.ochraceus L.YS--LKE--DGSLGDLTVNNDVPTGAPY..VD---RGEYML..TLSYARE--GAAM.IT.KL.D.DV.NTWHSYEG-.S....MAI.SN..FL.VV.V

C.hirsutipalpus L.YS--LKE--DGSLGNLTVNNDVPTGAPY..VD---RGEYML..TLSYARE--GAAM.IT.KL.D.DV.NTWHSYEG-.S....MAITSN..FL.VV.V

T.trivittatus L.YS--LKD--DNSLGELI.NNDVLAGFPY..AS---KGEYM...TLASAE.--VTAM.ITTKLND.SL.NIWHSYEG-.S....MAL.SN..FL.VA.V

L.abdullahbayrami L.YL--VEN--NDSFGKLSS.YDVPTGA.Y..A.---AGEYM...TLSYAR.--GGAM.IT.EVNN.N..NTWHSHKG-.SI....AI.SN..IL.VV.V

M.martensii L.YS--VEN--NDSFGKLSLNYDVPTGA.Y..A.---AGEYM...TLSYAR.--GGAM.IT.KMNN.N..NTWHSHKG-.S.....AI.SN..IL.VV.V

H.aztecus L.YR--V.SSRNDTAVMATSTFNVYLGA.Y..DH---KDNL....T.PKSQS--SDA..LTIDLQ.KKMLNIWNS.EG-.S.....AV.S..NFL.VV..

H.concolorus L.YR--V.SSRNDTAVMATSTFNIYLGA.Y..DH---KDNL....I.PKSQS--SDA..LTIDLQ.KKMLNIWNS.EG-.S.....AV.P..NFL.VV..

M.gertschi L.YP--V.SSRNDTAI.ATTTFNVYLGA.Y..AH---KDNL....T.PKSQS--SDAR.LTIDLDNKKM.N.WSSMEG-.V.....AV.P..NYL.VA..

C.coahuilae L.YQ--V.SFKNDT.ATAKTVFKV.LGA.Y..DH---KDNL....T.PKSQS--SDAY.LTIDLN.EKMLN.WSSLQG-.F.....AV.P..KSL.VA..

T.cristimanus L.YQ--V.SFRNDTNATVKTAFNV.LGH.Y..DH---KDNL....T.PKSQS--SDAH.LTIDLNRDKMVN.WSS.QG-.F.....AV.P..KSL.VA..

P.schwenkmeyeri L.YQ--V.SSRNYTAATAKTVFTV.LGR.Y..DH---KDNL....T.PKSQS--SDAH.LTIDLNREKMLN.WSS.QG-.F.....AV.P..KSL.VA..

S.gertschi L.YP--V.SSRNDTAA.ATTMFNV.LGA.Y..DH---KDNL....T.PKSPS--SDAH.LTIDLN.KKMLN.WSS.QG-.F.....AV.P..KSL.VA..

A.pococki bajae L.YP--V.SSRNDTAAIATTTFNM.LGA.Y..DH---KDNL....T.PKSQS--SDA..LTIDLN.KK..NIWSS.QG-.F.....AI.P..NSL.VA..

D.melici L.YP--V.SSTNDTGTTATTVFNVLLGP.Y.VDA---KDNH....T.PKSQ.--YVAR.LTIDLN..KLLNIWTSPLG-.S....LAV.P..SFL.VA..

U.yaschenkoi L.YP--V.SSRNNTGA.ATTAFNIILGP.Y..DD---KGNR....T.PKHQ.--FDA..LTIDLN..KLLNIWNSAQG-.F.....AV.P..NFL.VA..

**410 420**

**....|....|....|....|...**

**R.norvegicus_** H----TNTVWKFTLTEKMEH---

*pal*-PAM

PAL*m*

C.sculpturatus M----P.K....L---.VT.KST

C.hentzi M----P.K....L---.VT.KST

C.noxius M----P.K....L---.VT.KST

C.limpidus M----P.K....L---.VT.KST

C.orizaba M----P.K....L---.VT.KST

C.ochraceus M----P.K....L---.VT.KST

T.trivittatus G----P.KI...L---.VT.KST

L.abdullahbayrami G----P.K....L---.VT.KST

M.martensii G----P.K....L---.VT.KST

H.aztecus G----P.K....L---RDS.KST

H.concolorus G----P.K....L---RDS.KST

M.gertschi G----P.K....L---RDS.KSK

U.yaschenkoi G----P.K....L---RDS.KST

P.imperator G----P.R....L---RDS.KST

D.melici G----P.K....L---RDS.KST

T.cristimanus G----P.K....L---RDS.KST

C.coahuilae* G----P.K....L---RDS.KST

**D.melanogaster** GTNHQ..R...YV.A--------

C.sculpturatus G-KFTAKK.F..EINDVI-----

C.hentzi G-KFTAKK.F..EINDVI-----

C.noxius G-KFTAKK.F..EINDV------

C.limpidus G-KFTAKK.F..EINDVI-----

C.orizaba G-KFTSKK.F..EINDVI-----

C.ochraceus G-KFTSKK.F..EINDVI-----

C.hirsutipalpus G-KFTSKK.F..EINDV------

T.trivittatus G-KITPKKLF..KI.D-------

L.abdullahbayrami D-EFAPKKLF..LVSM-------

M.martensii G-EFAPKKLF..LVFDVI-----

H.aztecus E-IEAQRKLS..Q.LR-------

H.concolorus E-VEAQRKLS..Q.FR-------

M.gertschi G-NEAQRKLS..R.LK-------

C.coahuilae G-VEAQRKLS..R.LK-------

T.cristimanus G-VEAQRKLS..R.LK-------

P.schwenkmeyeri G-VEAQRKLS..R.LK-------

S.gertschi G-IEAQKKLS..R.LK-------

A.pococki bajae G-IEAQRKLS-------------

D.melici A-KDAPEQLS..Q.FQ-------

U.yaschenkoi G-KEAPKKLS..Q.L--------

**Supplementary Figure S6**. Sequence alignment of PAL domains. Sequences of scorpion *pal*-PAM and PAL*m* domains are aligned with sequences of *pal*-PAM from *Rattus norvegicus* (Uniprot:P14925) and PAL2 from *Drosophila melanogaster* (Uniprot:Q9V5E1). Color codes are as follows: residues involved in zinc coordination (H^585^, H^690^ and H^786^); residues involved in calcium coordination (V^520^, L^587^ and D^787^); cysteines involved in disulfide bridge formation (C); residues relevant for catalytic activity (Y^654^, R^706^); a tryptophan in a hydrophobic pocket for substrate binding (W^538^); N-glycosylation site (N^765^). Amino acid numbers correspond to the PAM1 isoform from *R. norvegicus*
