# Supplementary material for: The Dual α-Amidation System in Scorpion Venom Glands
Source: Toxins (Basel). 2019 Jul 20;11(7):425. doi: 10.3390/toxins11070425 (PMC6669573; doi:10.3390/toxins11070425)
Supplement: Supplementary file 1 [file toxins-11-00425-s001.zip › Delgado-Prudencio_Scorpion dual alpha-amidation system_Suplementary_Table_S2_R1.docx]

**Supplementary Table S2.** Sequence conservation between the catalytic domains of the bifunctional and monofunctional enzymes by species (% of identity). Only completely sequenced domains are reported.

|  |  | % of identity | |
| --- | --- | --- | --- |
| **Family** | **Species** | ***phm*-PAM / PHM*m*** | ***pal*-PAM / PAL*m*** |
| Buthidae | *Centruroides sculpturatus* | 29.5 | 32.5 |
|  | *Centruroides hentzi* | 30.4 | 32.8 |
|  | *Centruroides noxius* | 29.8 | 32.5 |
|  | *Centruroides limpidus* | 29.5 | 32.5 |
|  | *Centruroides orizaba* | 29.5 | 32.3 |
|  | *Centruroides ochraceus* | 30.1 | 32.5 |
|  | *Centruroides hirsutipalpus* |  |  |
|  | *Tityus trivittatus* | 29.0 | 32.0 |
|  | *Leiurus abdullahbayrami* | 29.5 | 31.5 |
|  | *Mesobuthus martensii* | 30.3 | 31.5 |
| Vaejovidae | *Thorellius cristimanus* |  | 31.7 |
|  | *Paravaejovis schwenkmeyeri* |  |  |
|  | *Chihuahuanus coahuilae* |  |  |
|  | *Serradigitus gertschi* |  |  |
| Caraboctonidae | *Hoffmannihadrurus aztecus* | 29.2 | 34.6 |
|  | *Hadrurus concolorus* | 29.7 | 34.2 |
| Euscorpiidae | *Megacormus gertschi* | 26.5 | 33.7 |
| Chactidae | *Anuroctonus pococki bajae* |  |  |
| Superstitionidae | *Superstitionia donensis* |  |  |
| Diplocentridae | *Diplocentrus melici* |  | 31.9 |
| Urodacidae | *Urodacus yaschenkoi* | 28.8 | 32.1 |
| Scorpionidae | *Pandinus imperator* | 28.0 |  |
