# Supplementary material for: The Dual α-Amidation System in Scorpion Venom Glands
Source: Toxins (Basel). 2019 Jul 20;11(7):425. doi: 10.3390/toxins11070425 (PMC6669573; doi:10.3390/toxins11070425)
Supplement: Supplementary file 1 [file toxins-11-00425-s001.zip › Delgado-Prudencio_Scorpion dual alpha-amidation system_Suplementary_Table_S3_R1.docx]

**Supplementary Table S3.** External groups used for the phylogenetic reconstruction of the evolutionary history of the functional domains.

| **Order** | **Species** | **PAM** | **PHM** | **PAL2** | **SRA RUN** | **ENTRIES** | | |
| --- | --- | --- | --- | --- | --- | --- | --- | --- |
|  |  |  |  |  |  | **PAM** | **PHM** | **PAL2** |
| **Pseudoscorpiones** | *Haplochernes kraepelini* |  | ✓ |  | SRR1767661 |  |  |  |
|  | *Neobisium carcinoides* |  |  |  | SRR7293662 |  |  |  |
|  | *Synsphyronus apimelus* |  | ✓ |  | SRR1145733 |  |  |  |
| **Amblypygi** | *Damon variegatus* |  | ⬛ |  | SRR1145694 |  |  |  |
| **Uropygi** | *Mastigoproctus giganteus* | ⬛**-**⬛ | ⬛ |  | SRR1145698 |  |  |  |
| **Araneae** | *Liphistius malayanus* | ⬛**-**⬛ | ⬛ |  | SRR1145736 |  |  |  |
|  | *Frontinella communis* | ✓ | ⬛ | ⬛ | SRR1145739 |  |  |  |
|  | *Parasteatoda tepidariorum* | ⬛**-**⬛ | ⬛ | ⬛ |  | XM_016071460.1  XP_015926946.1 | XM_016059674.2  XP_015915160.1 | XM_016069021.1  XP_015924507.1 |
|  | *Leucauge venusta* | ✓ | ⬛ | ⬛ | SRR1145740 |  |  |  |
| **Opiliones** | *Siro boyerae* | ✓ | ⬛ | ⬛ | SRR1145699 |  |  |  |
|  | *Trogulus martensi* | ⬛**-**⬛ | ⬛ | ⬛ | SRR1145730 |  |  |  |
|  | *Pachylicus acutus* |  |  |  | SRR1146670 |  |  |  |
|  | *Leiobunum verrucosum* |  |  |  | SRR1145701 |  |  |  |
| **Solifugae** | *Eremobates sp.* | ✓ |  |  | SRR1146672 |  |  |  |
| **Ricinulei** | *Ricinoides atewa* | ✓ | ⬛ | ✓ | SRR1145743 |  |  |  |
| **Xiphosura** | *Limulus polyphemus* | ⬛**-**⬛ | ⬛ | ⬛ |  | XP_013780664.1  XM_013925210.2 | XP_013784032.1  XM_013928578.2 | XP_013780668.1  XM_013925214.2 |
| **Diptera** | *Drosophila melanogaster* |  | ⬛ | ⬛ |  |  | NM_057877.4 | NM_001299847.1 |
|  | *Drosophila simulans* |  | ⬛ | ⬛ |  |  |  | XM_002082790.2 |
| **Rodentia** | *Rattus norvegicus* | ⬛**-**⬛ |  |  |  | P14925*  NM_013000.2 |  |  |
| **Primates** | *Homo Sapiens* | ⬛**-**⬛ |  |  |  | P19021*  NM_000919.3 |  |  |

(⬛**-**⬛, ⬛ and ⬛) Complete PAM, PHM and PAL sequences; (⬛**-**⬛) PAM sequences with >90% of the estimated complete sequence determined; (✓) Partial sequences with <50% of the estimated complete sequence determined; Empty spaces indicate that the protein does not exist in the species (mammals and flies) or that they have not been reported; (*) Uniprot accession codes.
