# Supplementary figures and images for: The Dual α-Amidation System in Scorpion Venom Glands
Source: Toxins (Basel). 2019 Jul 20;11(7):425. doi: 10.3390/toxins11070425 (PMC6669573; doi:10.3390/toxins11070425)

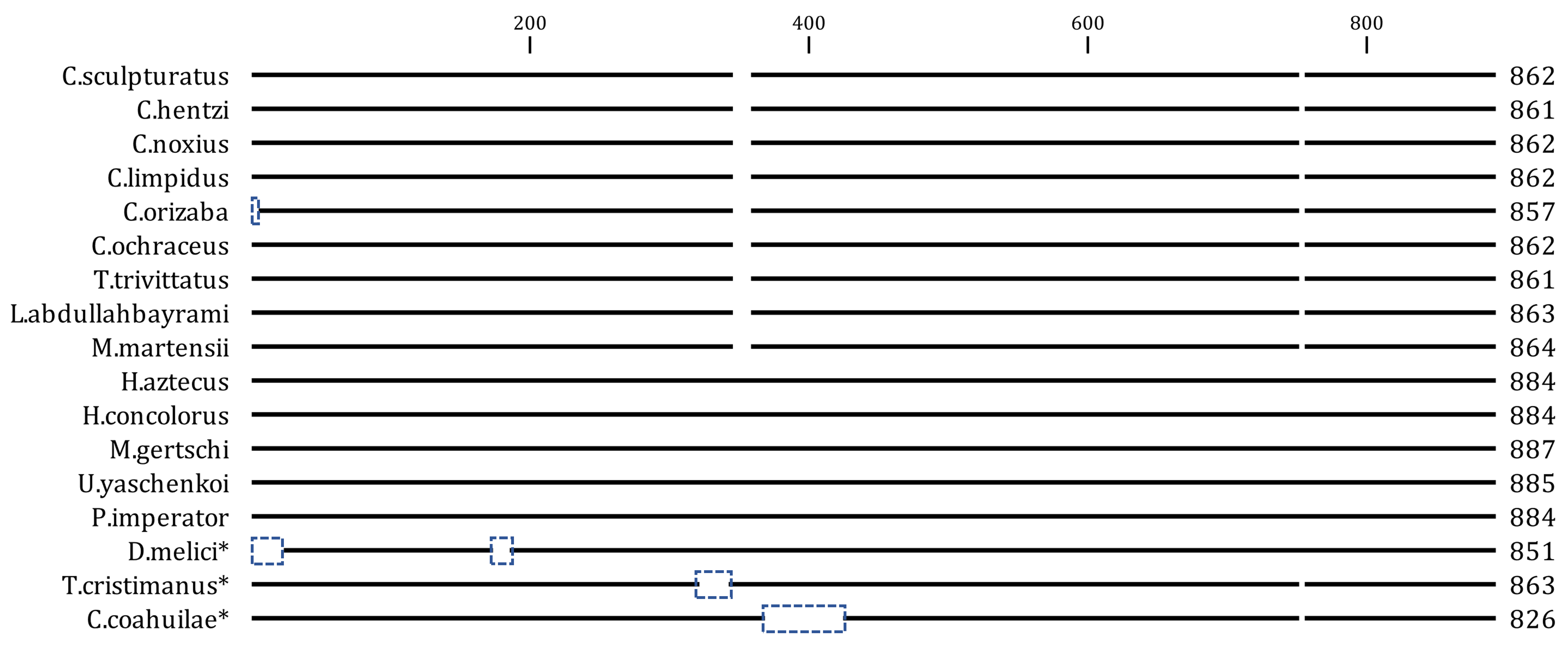

Supplement: Supplementary file 1 [file toxins-11-00425-s001.zip › Delgado-Prudencio_Scorpion dual alpha-amidation system_Supplementary_Figure_S1_R1.tif]

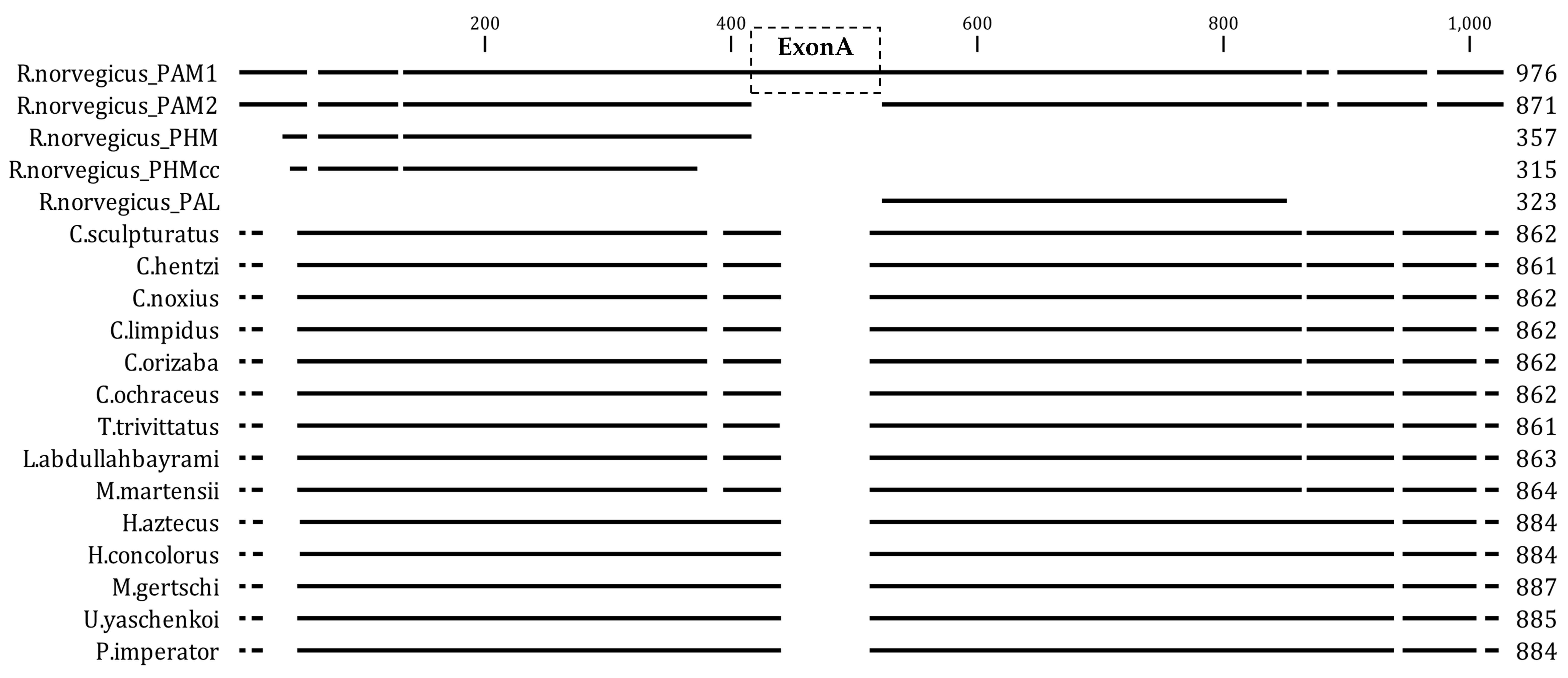

Supplement: Supplementary file 1 [file toxins-11-00425-s001.zip › Delgado-Prudencio_Scorpion dual alpha-amidation system_Supplementary_Figure_S2_R1.tif]

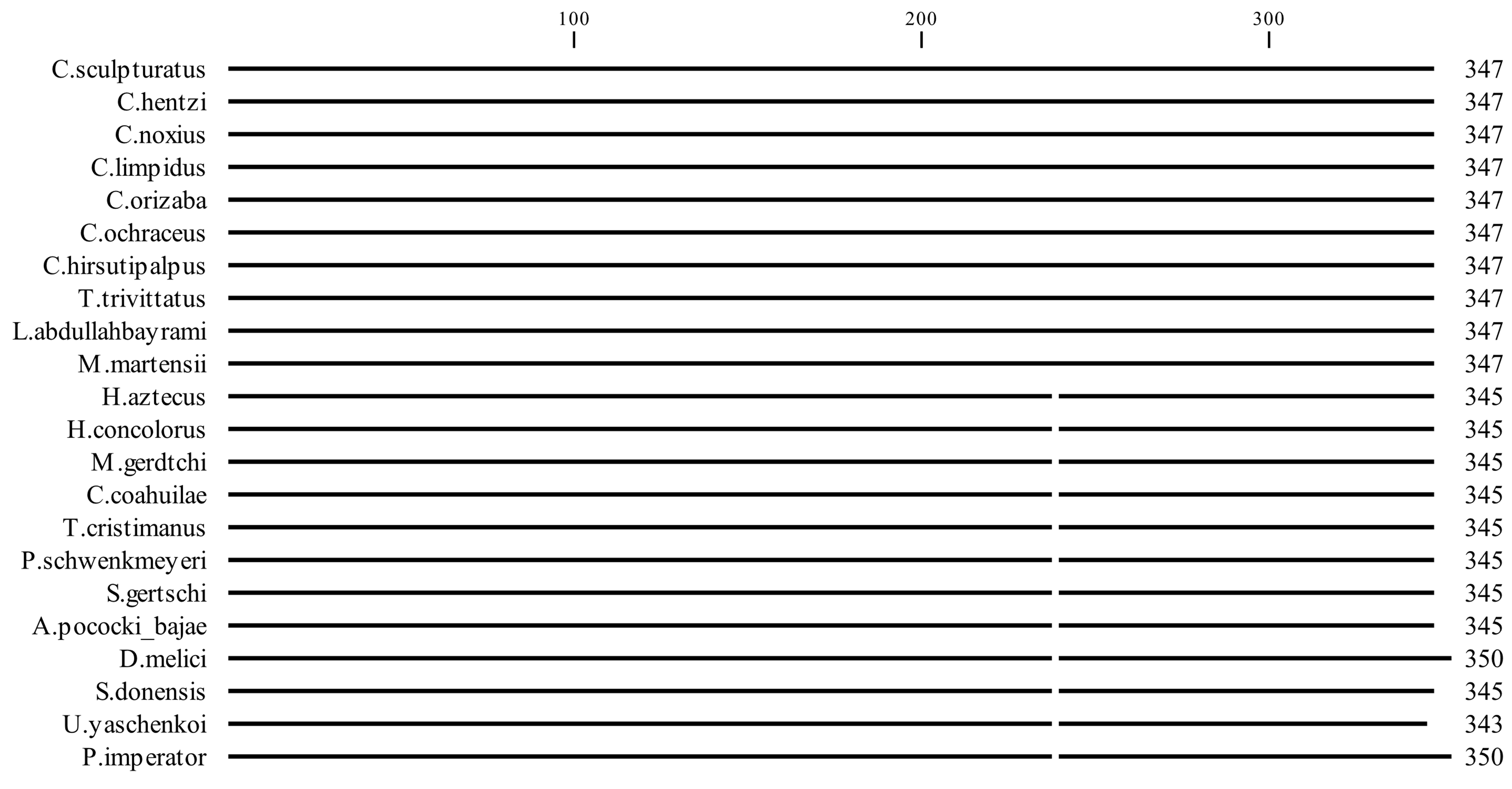

Supplement: Supplementary file 1 [file toxins-11-00425-s001.zip › Delgado-Prudencio_Scorpion dual alpha-amidation system_Supplementary_Figure_S3_R1.tif]

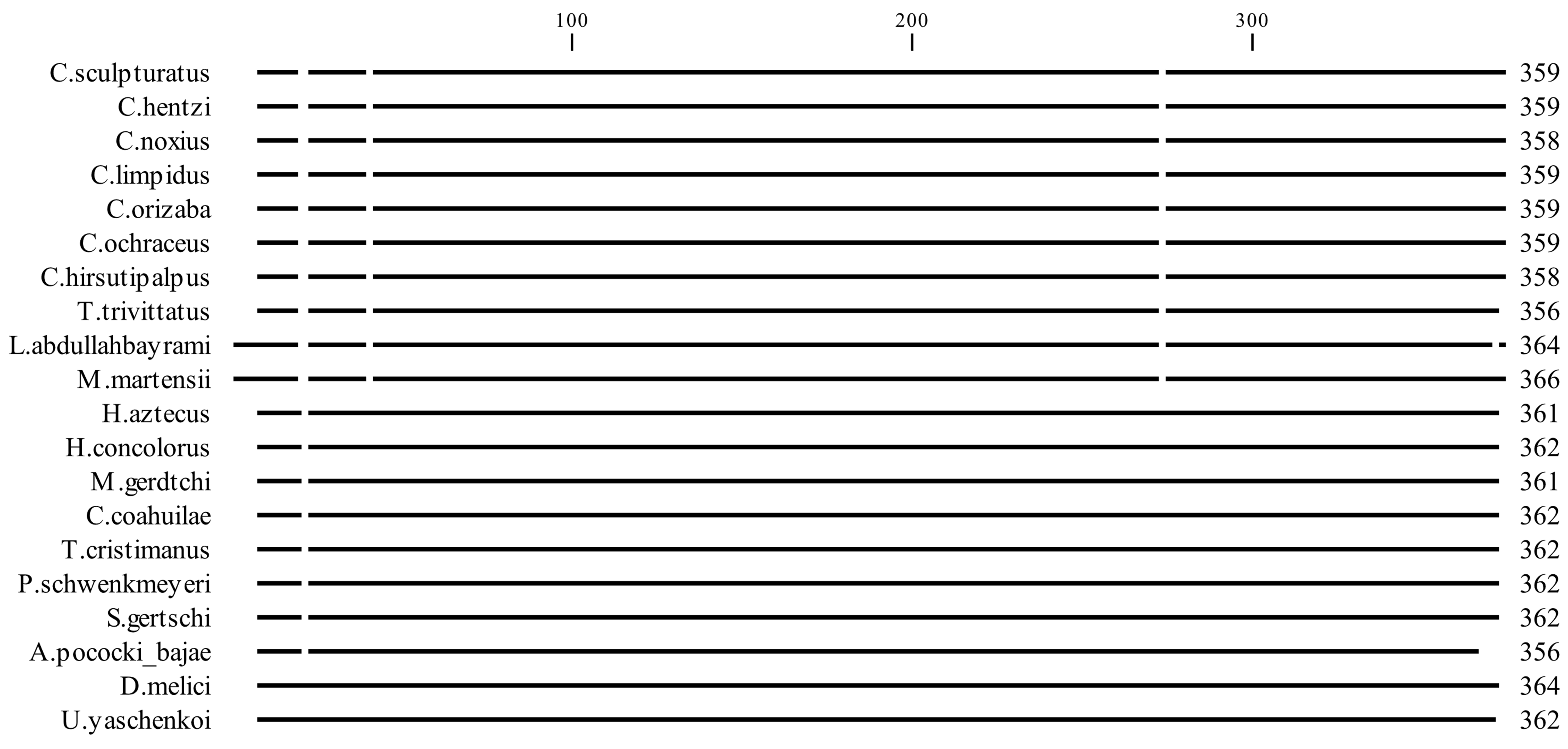

Supplement: Supplementary file 1 [file toxins-11-00425-s001.zip › Delgado-Prudencio_Scorpion dual alpha-amidation system_Supplementary_Figure_S4_R1.tif]
